# Causal relationship between type 1 diabetes mellitus and six high-frequency infectious diseases: A two-sample mendelian randomization study

**DOI:** 10.3389/fendo.2023.1135726

**Published:** 2023-03-31

**Authors:** Xiao-Hong Chen, Hong-Qiong Liu, Qiong Nie, Han Wang, Tao Xiang

**Affiliations:** ^1^ Emergency Department, The Affiliated Hospital of Southwest Jiaotong University, The Third People’s Hospital of Chengdu, Chengdu, Sichuan, China; ^2^ Department of Geriatrics, West China Hospital, Sichuan University, Chengdu, China; ^3^ Department of Geriatrics, The Affiliated Hospital of Southwest Jiaotong University, The Third People’s Hospital of Chengdu, Chengdu, Sichuan, China; ^4^ Department of Cardiology, The Affiliated Hospital of Southwest Jiaotong University, The Third People’s Hospital of Chengdu, Chengdu, Sichuan, China

**Keywords:** mendelian randomization, infections, intestinal, sepsis, lower respiratory, pregnancy, skin, urinary tract

## Abstract

**Purpose:**

Type 1 diabetes mellitus (T1DM) is associated with different types of infections; however, studies on the causal relationship between T1DM and infectious diseases are lacking. Therefore, our study aimed to explore the causalities between T1DM and six high-frequency infections using a Mendelian randomization (MR) approach.

**Methods:**

Two-sample MR studies were conducted to explore the causalities between T1DM and six high-frequency infections: sepsis, acute lower respiratory infections (ALRIs), intestinal infections (IIs), infections of the genitourinary tract (GUTIs) in pregnancy, infections of the skin and subcutaneous tissues (SSTIs), and urinary tract infections (UTIs). Data on summary statistics for T1DM and infections were obtained from the European Bioinformatics Institute database, the United Kingdom Biobank, FinnGen biobank, and Medical Research Council Integrative Epidemiology Unit. All data obtained for summary statistics were from European countries. The inverse-variance weighted (IVW) method was employed as the main analysis. Considering the multiple comparisons, statistical significance was set at p< 0.008. If univariate MR analyses found a significant causal association, multivariable MR (MVMR) analyses were performed to adjust body mass index (BMI) and glycated hemoglobin (HbA1c). MVMR-IVW was performed as the primary analysis, and the least absolute shrinkage and selection operator (LASSO) regression and MVMR-Robust were performed as complementary analyses.

**Results:**

MR analysis showed that susceptibility to IIs increased in patients with T1DM by 6.09% using the IVW-fixed method [odds ratio (OR)=1.0609; 95% confidence interval (CI): 1.0281–1.0947, p=0.0002]. Results were still significant after multiple testing. Sensitivity analyses did not show any significant horizontal pleiotropy or heterogeneity. After adjusting for BMI and HbA1c, MVMR-IVW (OR=1.0942; 95% CI: 1.0666–1.1224, p<0.0001) showed significant outcomes that were consistent with those of LASSO regression and MVMR-Robust. However, no significant causal relationship was found between T1DM and sepsis susceptibility, ALRI susceptibility, GUTI susceptibility in pregnancy, SSTI susceptibility, and UTI susceptibility.

**Conclusions:**

Our MR analysis genetically predicted increased susceptibility to IIs in T1DM. However, no causality between T1DM and sepsis, ALRIs, GUTIs in pregnancy, SSTIs, or UTIs was found. Larger epidemiological and metagenomic studies are required to further investigate the observed associations between the susceptibility of certain infectious diseases with T1DM.

## 1 Introduction

Type 1 diabetes mellitus (T1DM) is a chronic autoimmune illness characterized by hyperglycemia caused by insulin deficiency due to the secondary loss of the pancreatic islet β­cells (1). T1DM, the most prevalent autoimmune disorder in children and teenagers, currently affects more than 500,000 children globally (2). Long-term hyperglycemia can destroy microvascular and macrovascular systems. Therefore, patients with T1DM may develop multiple organ or tissue diseases, such as cardiovascular disease, cerebrovascular disease, peripheral artery disease, sudden cardiac death, and cognitive dysfunction (2).

Infectious diseases are experienced several times in almost every human. Many people may experience sepsis, a high-mortality syndrome that develops with infection (3). Acute lower respiratory infection (ALRI) was the most common cause of death in children in 2019 and accounted for the deaths of 700,000 children (4). Urinary tract infection (UTI) is a common infectious disease in adults, with more than half of all women experiencing at least one UTI in their life; moreover, UTIs are associated with significantly increased mortality in elderly people (5). Infection of the genitourinary tract (GUTI) is the most common bacterial infection in pregnancy, and GUTIs may cause preterm birth (6). Infection of the skin and subcutaneous tissues (SSTI) is another common infectious disease that can progress to a life-threatening infection (7). A recent nationwide study in the United States found that intestinal infection (II) was one of the most common infectious diseases experienced by patients in hospital (8).

Infectious diseases cause a significant burden on families and society. Recently, there has been much debate regarding whether T1DM increases the risk of infections. Some studies have found that patients with diabetes mellitus may be more susceptible to infectious diseases (9–11). However, Liberatore et al. argued that there was no strong relationship between T1DM and the occurrence of infections (12), and other studies revealed that the relationship between T1DM and infections is bidirectional (13). Remarkably, these studies had small sample sizes and possible confounding variables. Some recent meta-analyses found a significant positive relationship between T1DM and different infectious diseases (14–16); however, most of the studies pooled in meta-analyses were case-control studies and case series, which cannot negate the possibility of reverse causality. Therefore, we cannot distinguish which is the cause and which is the effect in the relationship between T1DM and infectious diseases through meta-analyses. A recent review argued that patients with poorly controlled diabetes were immunocompromised and hence were at increased risk of experiencing infectious diseases and their complications (17).

Confirming a causal relationship between T1DM and infectious diseases is challenging due to the possibility of reverse causality and confounders (18). Therefore, randomized controlled trials (RCTs) are considered the gold standard for exploring exposure-outcome causality; however, conducting rigorous RCTs is challenging because of the restriction of the medical ethics committees and selection of participants, and the extrapolation of results is limited because of the strict requirements of population selection (19).

The Mendelian randomization (MR) method was used to infer causal associations between exposures and outcomes using single nucleotide polymorphisms (SNPs) as instrumental variables (IVs) (20). Variants were randomly allocated from parents to offspring at conception. Therefore, the MR method was not susceptible to confounding or reverse causation, similar to the random assignment in RCTs (20). In this study, a two-sample MR was performed to investigate the causality of T1DM with high-frequency infections.

## 2 Materials and methods

### 2.1 Study design and data sources

#### 2.1.1 Genetic association datasets for T1DM

Summary statistical data for T1DM, including 9,266 cases and 15,574 controls with European ancestry, came from the European Bioinformatics Institute (EBI) database (21) and can be extracted from the genome-wide association study (GWAS) datasets by “GWAS ID” (**Table 1**). The EBI database is an international, innovative, interdisciplinary, and open dataset in life sciences (22).

**Table 1:** Profiles of exposure and outcomes in GWAS datasets.

Outcome	GWAS ID	Consortium	Sample Size	Number of Cases	Number of Controls	Year	Adjustment	Population
sepsis	ieu-b-4980	UK Biobank	486,484	11,643	474,841	2021	BMI, COPD, fatigue, arteritis, vaginitis, urolithiasis, depression, immunocompromised, LIDI, ISD	European
ALRIs	finn-b-J10_LOWERINF	FinnGen biobank	218,792	10,103	208,689	2021		
IIs	finn-b-AB1_VIRAL_OTHER_INTEST_INFECTIONS	FinnGen biobank	201,463	4,165	197,298	2021		
GUTIs in pregnancy	finn-b-O15_PREG_GU_INFECT	FinnGen biobank	111,731	1,401	110,330	2021		
SSTIs	finn-b-L12_INFECT_SKIN	FinnGen biobank	218,792	10,343	208,449	2021		
UTIs	ukb-b-8814	MRC-IEU	463,010	5,447	457,563	2018		
**Exposure**	**GWAS ID**	**Consortium**	**Sample Size**	**Number of Cases**	**Number of Controls**	**Year**	**nSNP**	**Population**
T1DM	ebi-a-GCST010681	EBI database	24,840	9,266	15,574	2020	12,783,129	European

GWAS, genome-wide association study; ID, identity; nSNP, number of single nucleotide polymorphism; ALRIs, acute lower respiratory infections; IIs, intestinal infections; GUTIs, infections of genitourinary tract; SSTIs, infections of the skin and subcutaneous tissue; UTIs, urinary tract infections; T1DM, type 1 diabetes mellitus; BMI, body mass index; COPD, chronic obstructive pulmonary disease; LIDI, Long-standing illness, disability or infirmity; ISD, Inflammatory skin disease; UK, United Kingdom; MRC-IEU, Medical Research Council Integrative Epidemiology Unit; EBI, European Bioinformatics Institute.

#### 2.1.2 Genetic association datasets for six high-frequency infections

As ALRIs, IIs, GUTIs in pregnancy, SSTIs, and UTIs were the most common infectious diseases in children, women, pregnant women, and in-hospital patients, respectively, and all infectious diseases can progress to sepsis; therefore, sepsis, ALRIs, IIs, GUTIs in pregnancy, SSTIs, and UTIs were chosen as the outcomes in our study.

Summary-level GWAS results regarding sepsis were obtained from UK Biobank with adjustment for age and sex. UK Biobank is an open dataset that included more than 500,000 participants for health-related research (23). Summary-level GWAS of ALRIs, IIs, GUTIs, and SSTIs were obtained from the FinnGen biobank. The FinnGen biobank is a well-known open dataset and comprises the genotype and phenotype data from approximately 20,000 Finnish individuals (24). Summary-level GWAS of UTIs were obtained from the Medical Research Council Integrative Epidemiology Unit (MRC-IEU). The MRC-IEU is an openly available dataset that comprises the most advanced population health science research in the United Kingdom, utilizing genetics, population data, and experimental interventions to identify the etiology of chronic disease (25).

All patients and controls (of both sexes) included in these summary-level GWASs were mainly of European descent. The detailed diagnostic criteria and the methods used to recruit participants in these GWASs are available in the original publications. No significant sample overlap was evident between these GWAS datasets. The profiles of GWAS datasets of T1DM and infections are summarized in **Table 1**.

#### 2.1.3 Informed consent statement and ethics approval statement

Informed consent and ethics approval were unnecessary for this study as consent and ethics approvals have already been obtained in the previous induvial studies. Furthermore, all summary-level GWAS data were obtained from an open dataset (https://gwas.mrcieu.ac.uk/).

### 2.2 Instruments selection

**Figure 1:**
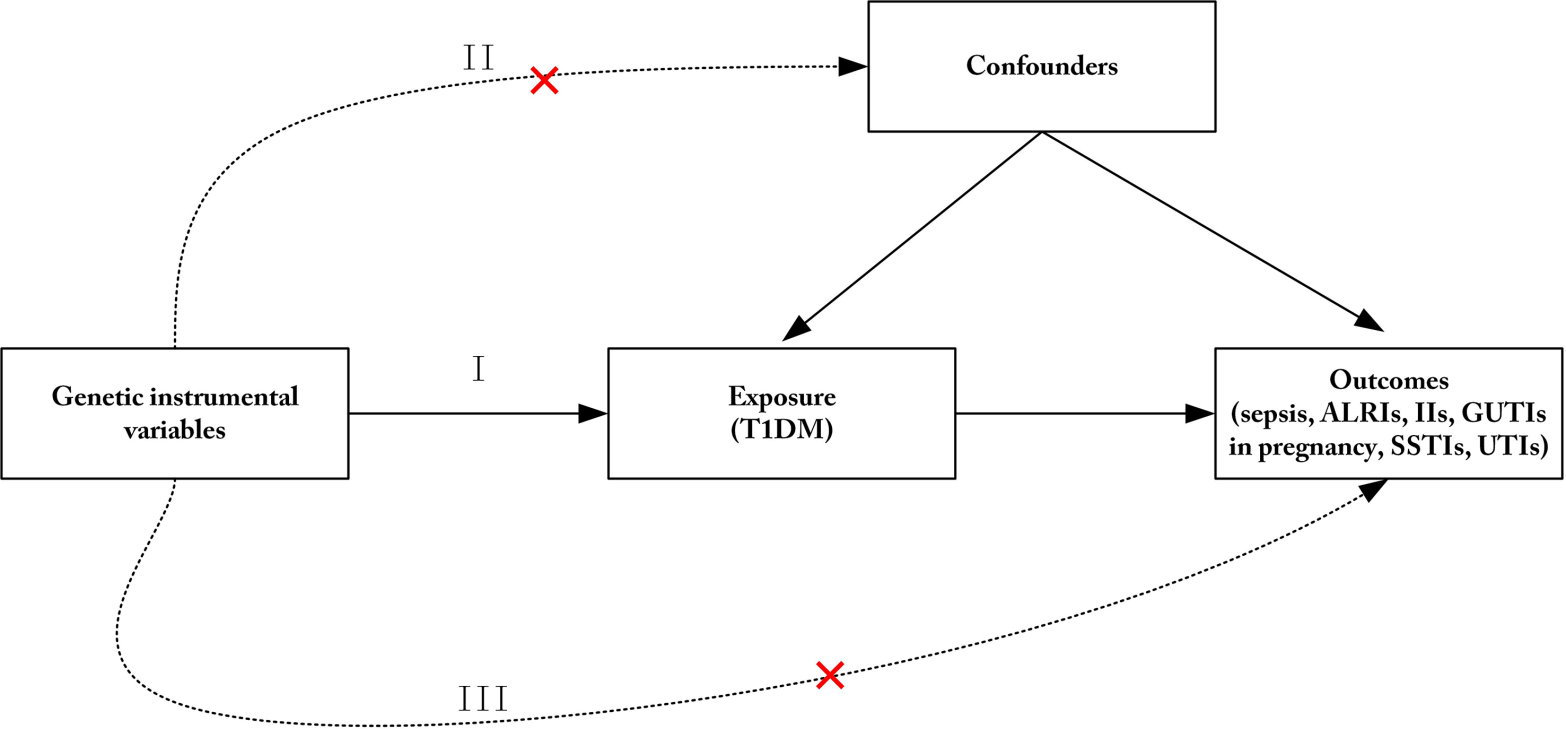
Three principles of the Mendelian randomization study. **(I)** The genetic instrumental variables (IVs) are strongly associated with T1DM; **(II)** The genetic IVs do not affect the outcome through the confounders; **(III)** The genetic IVs do not affect outcomes directly, but only *via* exposure. ALRIs, acute lower respiratory infections; IIs, intestinal infections; GUTIs, infections of the genitourinary tract; SSTIs, Infections of the skin and subcutaneous tissue; UTIs, urinary tract infections; T1DM, type 1 diabetes mellitus.

Three key assumptions for MR analysis should be met (**Figure 1**) (19). SNPs linked to T1DM at the genome-wide significance levels from a meta-analysis of GWAS were selected as IVs (p< 5  10^−8^). Additionally, the related linkage disequilibrium was considered (kb=10,000, r^2^<0.001) (26). Subsequently, F statistic was calculated to evaluate the strength between the IVs and exposure with the following equation: $F=\frac{N-k-1}{k}\times \frac{R^{2}}{1-R^{2}}$ (27), where R^2^ is the quantity that IVs can represent exposure, k is the number of SNPs selected, and N is the exposure sample size. If F is more than 10, the relationship is believed to be robust enough to minimize the bias induced by weak IVs. Thereafter, the SNPs related to potential confounders of the outcomes were eliminated. SNPs were selected after adjusting for body mass index, chronic obstructive pulmonary disease, fatigue, arteritis, vaginitis, urolithiasis, depression, immunocompromised, long-standing illness, disability or infirmity, and inflammatory skin disease, which may influence the risk of sepsis, ALRIs, IIs, GUTIs in pregnancy, SSTIs, and UTIs in PhenoScanner V2 (http://www.phenoscanner.medschl.cam.ac.uk/) (28). Finally, SNPs that were not directly associated with outcomes (sepsis, ALRIs, IIs, GUTIs in pregnancy, SSTIs, and UTIs) were selected using PhenoScanner V2 (2018 Cardiovascular Epidemiology Unit, University of Cambridge) (28).

We attempted to infer positive-strand alleles using allele frequencies for palindromes across exposure and outcome datasets. SNPs for incompatible alleles and palindromic sequences with intermediate allele frequencies were removed. Proxy SNPs were used when the SNPs were unavailable in the outcomes GWAS datasets, and the minimum linkage disequilibrium r^2^ was set at 0.8. Final IVs for ensuing MR analysis were then carefully selected.

### 2.3 MR analysis

The inverse-variance weighted (IVW) method was used as the main analysis approach because of its precise estimate (29). When heterogeneity was statistically significant, the random effect model was utilized. Otherwise, the fixed effect model was utilized. Other MR methods were also performed to guarantee the effectiveness and stability of the outcomes. The maximum likelihood method provides an estimator with the lowest standard error under essentially all conditions compared with other MR methods (30). The penalized weighted median (PWM) estimator is a novel MR method that can provide a consistent estimator when significant heterogeneity exists (31). The weighted median estimator (WME) can control type I errors efficiently to improve the detection ability of causal effects and can provide a stable estimator, even when more than 50% of the information is acquired from invalid IVs (32). The MR-Egger method can identify and correct potential pleiotropy and provide a relatively consistent estimate (33). Considering the multiple comparisons, the Bonferroni method was performed to rectify overall type I errors, and p<0.008 (α = 0.05/6) was considered statistically significant. If a significant causal association was found in univariate MR analyses, multivariable MR (MVMR) analyses would be performed. MVMR-IVW was performed as the primary analysis (34). The least absolute shrinkage and selection operator (LASSO) regression provides the best estimation for moderate-to-high levels of pleiotropy and valid inference in a three-sample setting (35). MVMR-Robust supplies precision estimates in all scenarios (even with 70% pleiotropy), corrects type I error rates, and assesses instrument strength (35). Therefore, we conducted LASSO regression and MVMR-Robust as complementary analyses. MVMR analyses were performed by adjusting for potential confounders, including body mass index (BMI), which had a causality association with T1DM and infectious diseases in previous MR studies (36, 37), and glycated hemoglobin (HbA1c), which reflects the average blood sugar over the past 3 months. We choose HbA1c as a confounder, as numerous studies have found that the status of hyperglycemia in diabetes was responsible for increased morbidity and mortality of infectious diseases rather than diabetes itself (17, 38). Detailed information on the genetic association datasets of the two confounders is presented in **Supplementary Table 1**.

### 2.4 Sensitivity analysis

Cochran’s Q statistic (R Foundation for Statistical Computing, Vienna, Austria) was used to identify SNP heterogeneity (34). The MR-Egger intercept (differs on average from zero) method was used to test whether genetic variants of T1DM have pleiotropic effects on infections (33). Mendelian randomization pleiotropy residual sum and outlier (MR-PRESSO) (39) and leave-one-out (40) methods were employed to detect potential outlier SNPs. The forest plot was used to exhibit the single SNP effect size of T1DM on infections. The scatter plot was used to test the causal effects of T1DM on infections. Finally, the funnel plot was used to demonstrate the symmetrical distribution of the selected SNPs. For sensitivity analysis, p<0.05 indicated statistical significance. All data were analyzed using the “TwoSampleMR,” “MRPRESSO,” “MendelianRandomization,” and “MVMR” packages in R software 4.0.5 (R Foundation for Statistical Computing).

## 3 Results

After the initial screening, 44 SNPs were obtained as IVs. Extensive information on 44 SNPs is presented in **Supplementary Table 2**. Five SNPs (rs9273363, rs1131017, rs6679677, rs10830227, and rs2111485), which may be associated with confounders, were deleted using the PhenoScanner tool (http://www.phenoscanner.medschl.cam.ac.uk/), and no SNP traits were associated with sepsis, ALRIs, IIs, GUTIs in pregnancy, SSTIs, and UTIs (**Table 2**). Finally, we obtained 39 SNPs as IVs of T1DM.

**Table 2:** Deleted SNPs associated with confounders.

confounders	SNPs
**BMI**	**rs9273363, rs1131017**
**COPD**	**-**
**Fatigue**	**-**
**Arteritis**	**-**
**Vaginitis**	**-**
**Urolithiasis**	**-**
**Depression**	**rs10830227**
**Immunocompromised**	**-**
**LIDI**	**rs6679677**
**ISD**	**rs2111485**

BMI, body mass index; COPD, chronic obstructive pulmonary disease; LIDI, long-standing illness, disability or infirmity; ISD, inflammatory skin disease; SNP, single nucleotide polymorphism.

### 3.1 MR Analysis Between T1DM and IIs

#### 3.1.1 Univariate MR analyses

After removing SNP (rs34954) for incompatible alleles, we did not find proxy SNP in the GWAS dataset of IIs. Finally, we obtained 33 SNPs as IVs in the MR analysis of T1DM on IIs. For these IVs, the F statistic was 22.80604. Detailed information about the SNPs of T1DM on IIs is shown in **Supplementary Table 3**.

The risk of IIs was found to be increased in patients with T1DM by 6.09% using the IVW method (OR=1.0609; 95% CI 1.0281–1.0947, p=0.0002), increased by 7.09% using the MR-Egger method (OR=1.0709; 95% CI, 1.0161–1.1286; p=0.0158), increased by 6.35% using the WME method (OR=1.0635; 95% CI, 1.0143–1.115; p=0.0108), increased by 6.12% using the Maximum likelihood method (OR=1.0612; 95% CI, 1.0281–1.0954; p=0.0002), and increased by 6.12% using the PWM method (OR=1.0634; 95% CI, 1.0141–1.115; p=0.0112). The results are shown in **Table 3**.

**Table 3:** Results of main MR analyses on the causal effects of T1DM with six infections.

Outcome	MR method	SNPs No.	OR (95% CI)	SE	p-value
Sepsis	IVW-random	38	0.9926 (0.9727, 1.0129)	0.0103	0.4709
	IVW-fixed	38	0.9926 (0.9754, 1.0101)	0.0089	0.4047
	MR-Egger	38	0.9881 (0.9543, 1.0231)	0.0178	0.5051
	WME	38	0.9988 (0.9734, 1.0249)	0.0131	0.9293
	Maximum likelihood	38	0.9925 (0.9752, 1.0101)	0.0090	0.4023
	PWM	38	0.9989 (0.9731, 1.0253)	0.0133	0.9314
ALRIs	IVW-random	33	1.0181 (0.9977, 1.0390)	0.0104	0.0831
	IVW-fixed	33	1.0181 (0.9977, 1.0390)	0.0104	0.0831
	MR-Egger	33	1.0162 (0.9829, 1.0507)	0.0170	0.3509
	WME	33	1.0070 (0.9792, 1.0355)	0.0142	0.6261
	Maximum likelihood	33	1.0182 (0.9977, 1.0392)	0.0104	0.0825
	PWM	33	1.0070 (0.9784, 1.0364)	0.0147	0.6360
**IIs**	IVW-random	33	1.0609 (1.0279, 1.0950)	0.0161	**0.0002**
	IVW-fixed	33	1.0609 (1.0281, 1.0947)	0.0160	**0.0002**
	MR-Egger	33	1.0709 (1.0161, 1.1286)	0.0268	**0.0158**
	WME	33	1.0635 (1.0143, 1.1150)	0.0241	**0.0108**
	Maximum likelihood	33	1.0612 (1.0281, 1.0954)	0.0162	**0.0002**
	PWM	33	1.0634 (1.0141, 1.1150)	0.0242	**0.0112**
GUTIs in pregnancy	IVW-random	33	0.9889 (0.9378, 1.0427)	0.0270	0.6791
	IVW-fixed	33	0.9889 (0.9378, 1.0427)	0.0270	0.6791
	MR-Egger	33	1.0196 (0.9345, 1.1124)	0.0444	0.6655
	WME	33	1.0033 (0.9294, 1.0832)	0.0391	0.9323
	Maximum likelihood	33	0.9887 (0.9374, 1.0429)	0.0272	0.6772
	PWM	33	1.0030 (0.9271, 1.0851)	0.0401	0.9404
SSTIs	IVW-random	33	1.0036 (0.9788, 1.0290)	0.0128	0.7794
	IVW-fixed	33	1.0036 (0.9833, 1.0243)	0.0104	0.7312
	MR-Egger	33	0.9923 (0.9520, 1.0343)	0.0211	0.7167
	WME	33	1.0185 (0.9892, 1.0486)	0.0149	0.2186
	Maximum likelihood	33	1.0037 (0.9832, 1.0245)	0.0105	0.7286
	PWM	33	1.0188 (0.9883, 1.0502)	0.0155	0.2309
UTIs	IVW-random	26	0.9999 (0.9996, 1.0003)	0.0002	0.7487
	IVW-fixed	26	0.9999 (0.9996, 1.0003)	0.0002	0.7356
	MR-Egger	26	1.0000 (0.9994, 1.0005)	0.0003	0.8692
	WME	26	0.9999 (0.9995, 1.0003)	0.0002	0.6085
	Maximum likelihood	26	0.9999 (0.9996, 1.0003)	0.0002	0.7346
	PWM	26	1.0000 (0.9994, 1.0003)	0.0002	0.6245

T1DM, Type 1 diabetes mellitus; IVW, inverse-variance weighted; WME, weighted median estimator; PWM, penalized weighted median; No., number of; SE, standard error; ALRIs, acute lower respiratory infections; IIs, intestinal infections; GUTIs, infections of genitourinary tract; SSTIs, Infections of the skin and subcutaneous tissue; UTIs, urinary tract infections; OR, odds ratio; CI, confidence interval. Statistical significance was set at p< 0.05.

**Figure 2:**
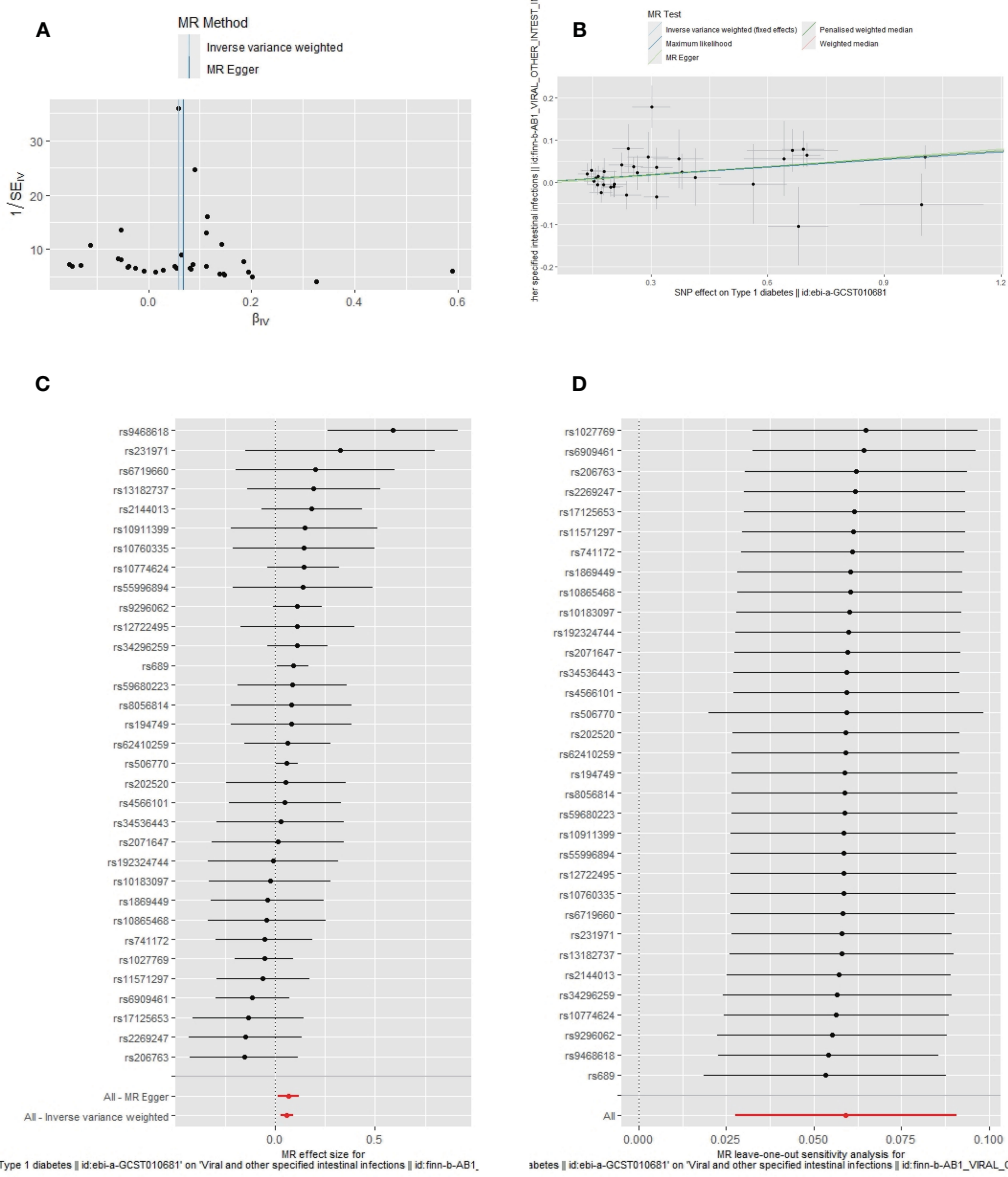
The funnel plot **(A)**, scatter plot **(B)**, Forest plot **(C)** and leave-one-out plot **(D)** of the genetic risk of T1DM on IIs.

No heterogeneity (MR-Egger, p=0.4049; IVW, p=0.4447) and no potential pleiotropy (MR-Egger, intercept=-0.0041; p=0.6633) were observed in the univariate MR analysis. The MR-PRESSO test showed no outliers. The funnel plot showed the selected SNPs were distributed symmetrically (**Figure 2A**). The scatter plot demonstrated the causality between T1DM and IIs (**Figure 2B**). The forest plot displays the effect size for every single SNP on the risk of IIs and shows that causality existed between T1DM and the occurrence of IIs (**Figure 2C**). The “Leave-one-out plot” suggested that no SNP had an important impact on the estimated causal association (**Figure 2D**).

#### 3.1.2 MVMR analyses

The MVMR-IVW estimates also showed that T1DM had a significant causal association with IIs after adjusting for BMI and HbA1c (OR=1.0942; 95% CI, 1.0666–1.1224; p<0.0001). No heterogeneity (MR-IVW, p=0.9580) was observed. The LASSO regression (OR=1.0942; 95% CI, 1.0666 - 1.1224; p<0.001) and MVMR-Robust (MVMR-Robust, OR= 1.0932; 95% CI, 1.0608 - 1.1263; p<0.05) results were consistent with those of the MVMR-IVW analyses. The F-statistic was 45.4855, and the number of valid instruments was 88.

### 3.2 MR analysis between T1DM and sepsis, ALRIs, GUTIs in pregnancy, SSTIs, and UTIs

One SNP (rs34954) was removed for incompatible alleles when combining the GWAS data of sepsis, ALRIs, GUTIs in pregnancy, and SSTIs separately with the selected IVs of T1DM. No SNP was removed for incompatible alleles when combining the GWAS data of UTIs and the selected IVs of T1DM. No proxy SNP was found in the GWAS dataset of ALRIs, GUTIs in pregnancy, SSTIs, and UTIs. SNP (rs34296259) was proxied by rs190824943 in the GWAS dataset of sepsis. Finally, we obtained 38, 33, 33, 33, and 26 SNPs, respectively, as IVs in the MR analysis of T1DM on sepsis, ALRIs, GUTIs in pregnancy, SSTIs, and UTIs separately. For these selected IVs, F statistics were more than 10. Detailed information about SNPs of T1DM on sepsis, ALRIs, GUTIs in pregnancy, SSTIs, and UTIs are separately presented in **Supplementary Tables 4**-**8**.

The primary IVW-fixed method showed no causality between T1DM and sepsis, ALRIs, GUTIs in pregnancy, SSTIs, or UTIs. The other MR method (MR-Egger, WME, maximum likelihood, and PWM) results were consistent with IVWs (**Table 3**).

**Figure 3:**
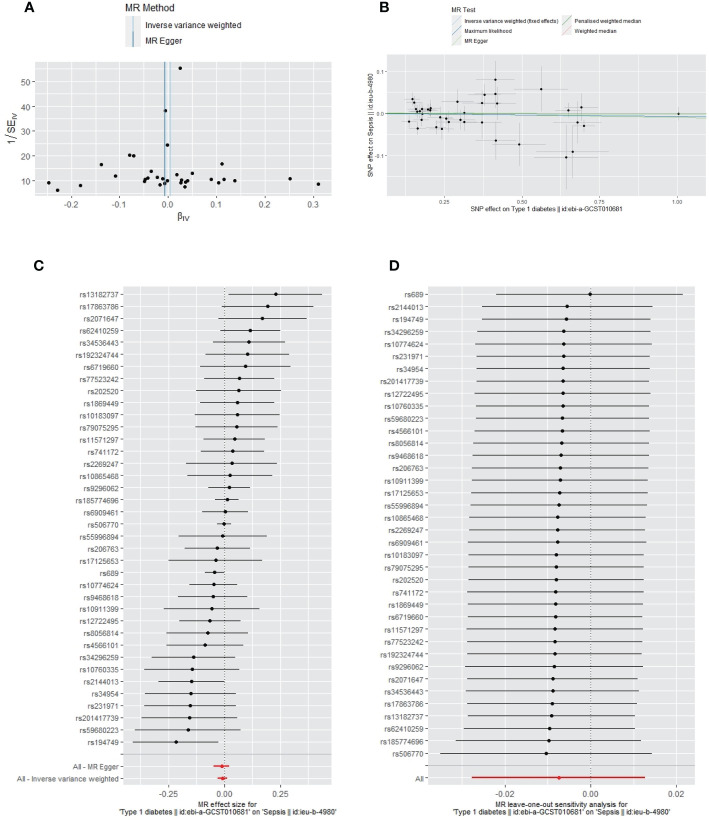
The funnel plot **(A)**, scatter plot **(B)**, Forest plot **(C)** and leave-one-out plot **(D)** of the genetic risk of T1DM on sepsis.

**Figure 4:**
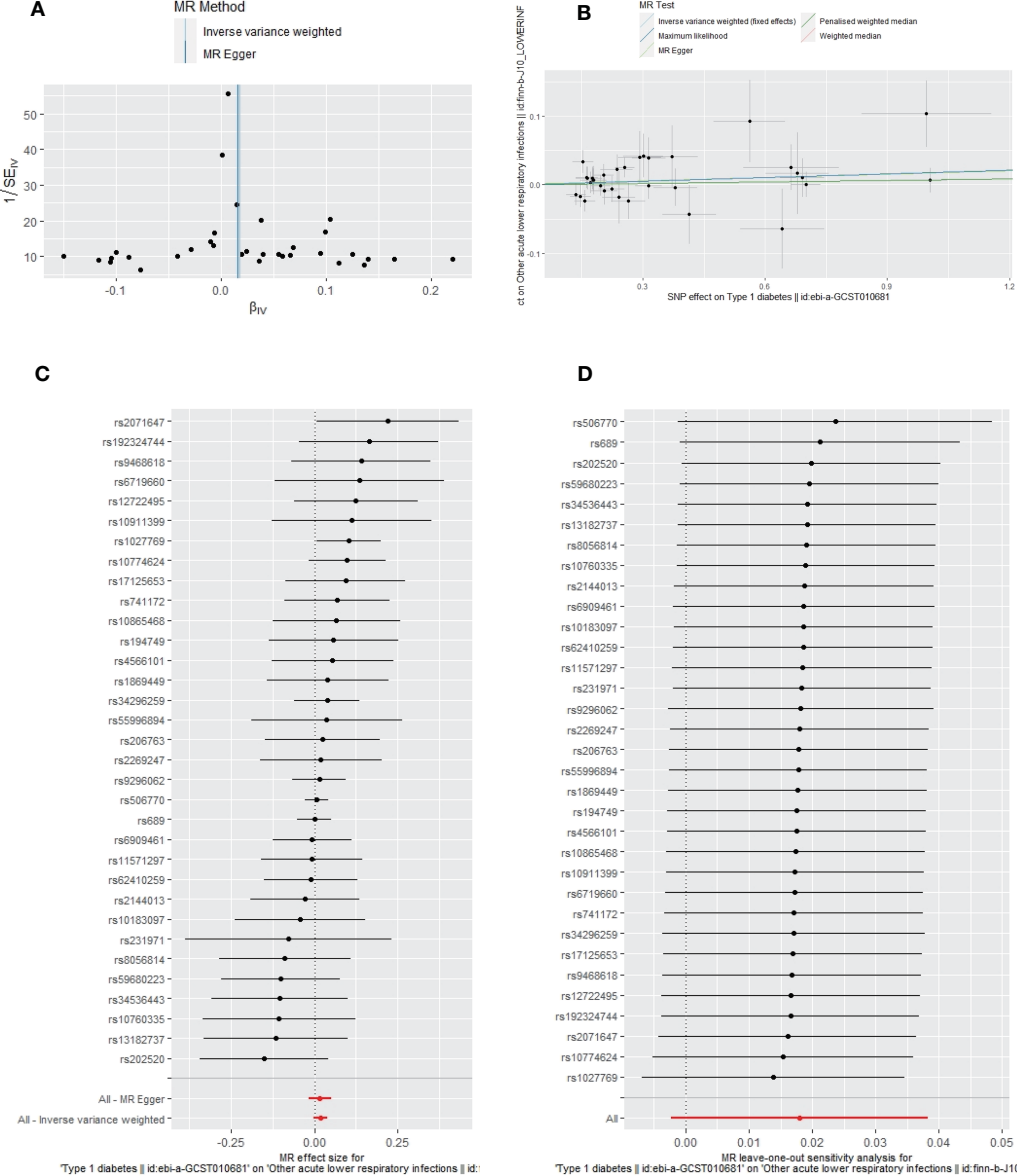
The funnel plot **(A)**, scatter plot **(B)**, Forest plot **(C)** and leave-one-out plot **(D)** of the genetic risk of T1DM on ALRIs.

**Figure 5:**
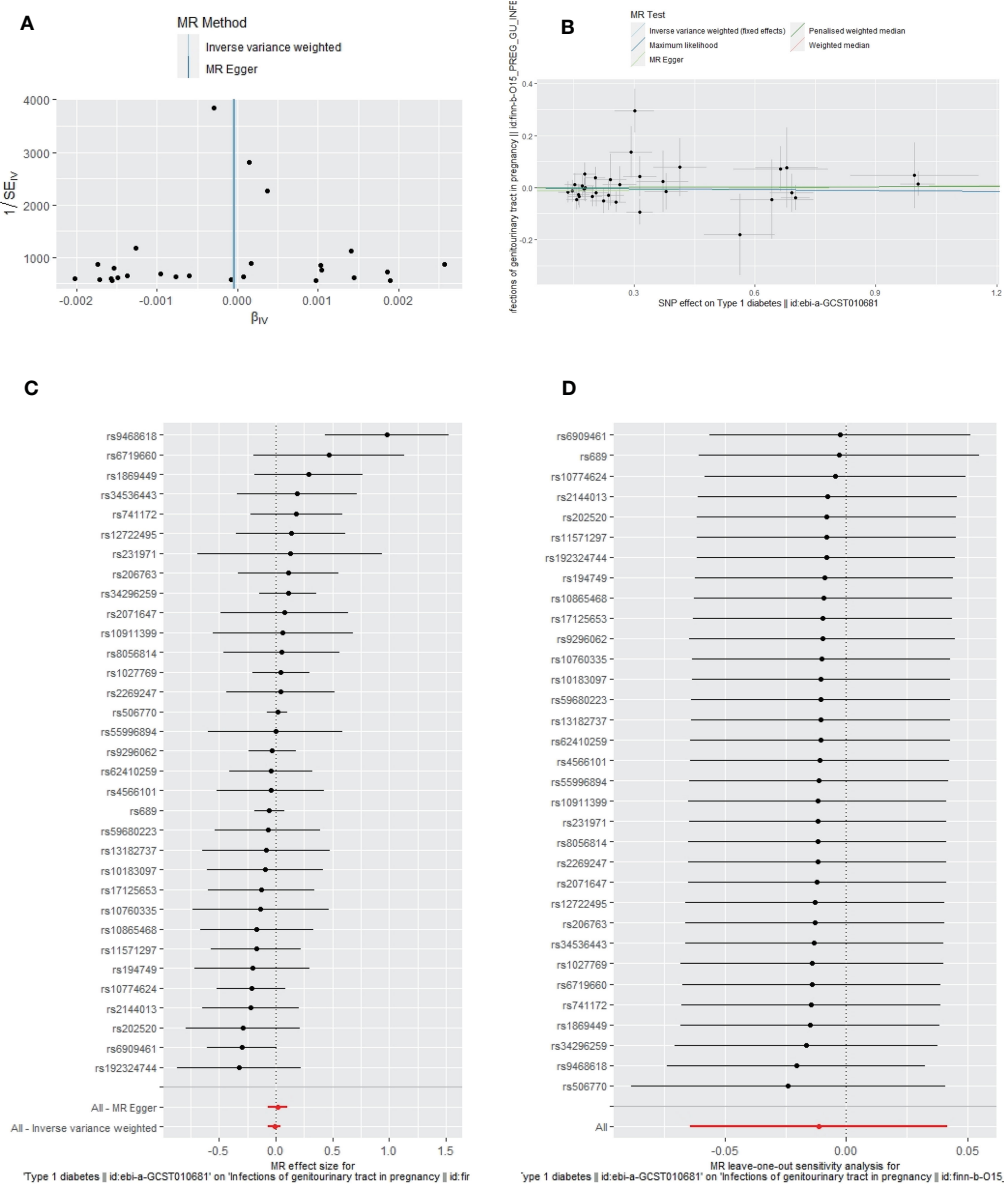
The funnel plot **(A)**, scatter plot **(B)**, Forest plot **(C)** and leave-one-out plot **(D)** of the genetic risk of T1DM on GUTIs in pregnancy.

**Figure 6:**
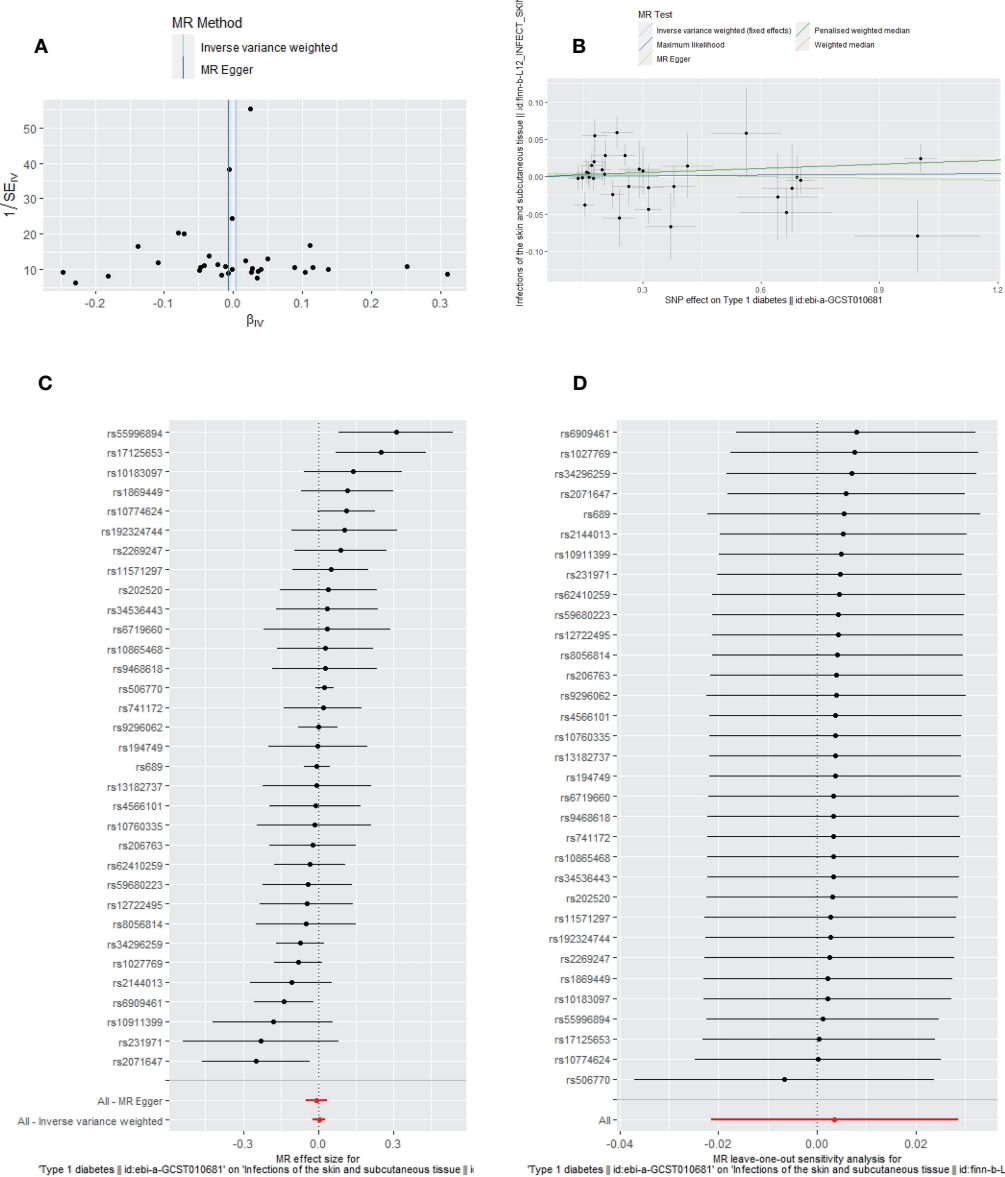
The funnel plot **(A)**, scatter plot **(B)**, Forest plot **(C)** and leave-one-out plot **(D)** of the genetic risk of T1DM on SSTIs.

**Figure 7:**
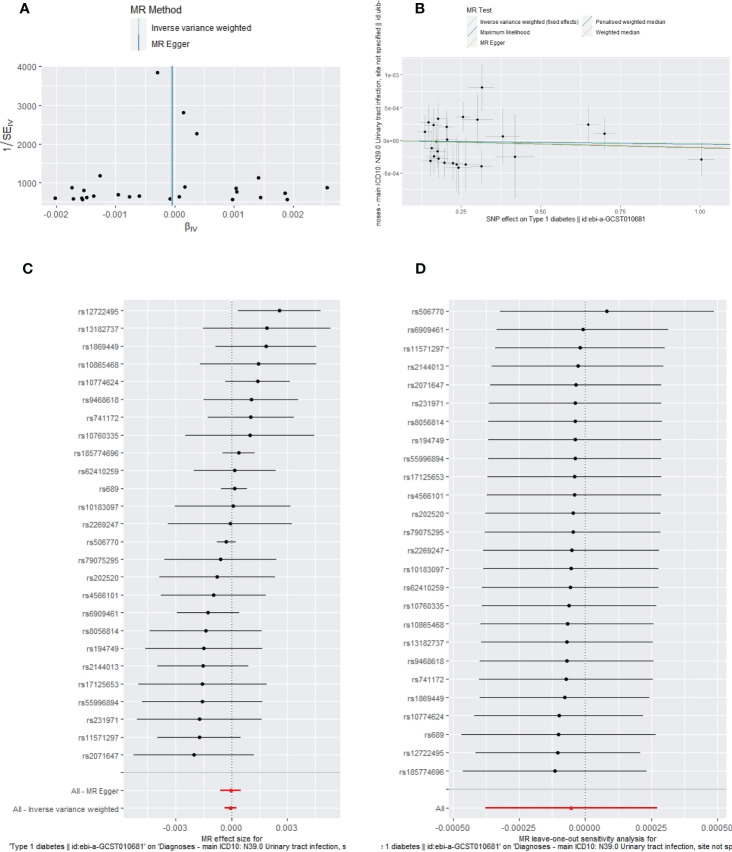
The funnel plot **(A)**, scatter plot **(B)**, Forest plot **(C)** and leave-one-out plot **(D)** of the genetic risk of T1DM on UTIs.

Apart from a significant heterogeneity in the analysis of T1DM on SSTIs, no other heterogeneity was observed in the analysis of T1DM on sepsis, ALRIs, GUTIs in pregnancy, or UTIs. No potential pleiotropy or outliers were observed in the analysis of T1DM on sepsis, ALRIs, GUTIs in pregnancy, UTIs, and SSTIs (**Table 4**). The funnel plots, the scatter plots, the forest plots, and the leave-one-out plots of T1DM for sepsis, ALRIs, GUTIs in pregnancy, SSTIs, and UTIs are displayed in **Figures 3**–**7** separately.

**Table 4:** Sensitive analysis of T1DM on outcomes.

Outcomes	F statistics	Heterogeneity		Pleiotropy (MR-Egger)		Outliers (MR-PRESSO)
		MR-Egger	IVW	Intercept	p-value	
**Sepsis**	22.27958	p=0.0691	p=0.0833	0.002	0.7546	NO
**ALRIs**	22.79959	p=0.6079	p=0.6557	0.0008	0.8921	NO
**IIs**	22.80604	p=0.4049	p=0.4447	-0.0041	0.6633	NO
**GUTIs in pregnancy**	22.87157	p=0.5019	p=0.5144	-0.0134	0.3923	NO
**SSTIs**	22.79959	p=0.0299	p=0.0336	0.005	0.5048	NO
**UTIs**	30.5624	p=0.2689	p=0.3174	-2.882E-6	p =0.9769	NO

T1DM, Type 1 diabetes mellitus; IVW, inverse-variance weighted; MR-PRESSO, Mendelian randomization pleiotropy residual sum and outlier; ALRIs, acute lower respiratory infections; IIs, intestinal infections; GUTIs, infections of genitourinary tract; SSTIs, Infections of the skin and subcutaneous tissue; UTIs, urinary tract infections. Statistical significance was set at p< 0.05.

## 4 Discussion

Based on the first MR study, we investigated the causality of T1DM in six high-frequency infections. There were two main results of the present MR study. First, the risk of IIs increased by 6.09% (OR=1.0609; 95% CI: 1.0281–1.0947, p=0.0002) in patients with T1DM even when a significant threshold was set at 0.008 (corrected by the Bonferroni method). The result was confirmed by sensitivity analyses. In the MVMR analyses, after adjusting for BMI and HbA1c, the causality of T1DM on IIs was still significant (MVMR-IVW, OR=1.0942; 95% CI, 1.0666–1.1224; p<0.0001); these results were consistent with those of the LASSO regression and MVMR-Robust. Second, T1DM may not be responsible for some infections, such as sepsis, ALRIs, GUTIs in pregnancy, SSTIs, and UTIs.

The findings of the present MR study did not support the findings of some observational studies that suggested that patients with T1DM had an increased risk of contracting some infections (41–43). A prospective cohort study reported that patients with T1DM were at a highly increased risk of lower respiratory tract, urinary tract, and skin infection (42). Another retrospective study that adjusted for age and sex also found that patients with T1DM were more susceptible to infection with the SARS-CoV-2 virus (43). Hence, our findings are consistent with those of many previous observational studies (12, 44, 45). One case-control study concluded that patients with T1DM were not susceptible to infections by comparing the plasma levels of immunoglobulins and complement proteins in patients with T1DM and controls (12). A survey with 10 years of follow-up stated that sexual activity rather than the T1DM was linked to an increased risk of UTIs in women (44). Czaja et al. believed that patients with T1DM may have an increased risk of asymptomatic bacteriuria (ASB). Nevertheless, due to impaired immunity caused by T1DM, ASB would not progress to a symptomatic infectious disease (44). Another prospective study showed that the risk of sepsis increased when T1DM was complicated with hind foot ulceration (45). Recently, a meta-analysis discovered that diabetes mellitus did not affect sepsis prognosis, although high blood glucose levels did (46).

T1DM and infections have been linked bidirectionally. As mentioned above, some studies argued that T1DM increased the occurrence of infections, whereas many other studies found that infections also have a marked influence on the onset of T1DM. For example, a study based on a population-based registry in Abruzzo (central Italy) found that multiple bacterial infections before diabetes mellitus could significantly delay the onset of T1DM (47). A review summarized that enterovirus infections, such as coxsackievirus B4, accelerated or promoted the initiation of T1DM (48). Another review also suggested that gut dysbiosis might be associated with the onset of T1DM, and different intestinal bacteria play different roles (49).

T1DM is a chronic disease, and patients with this disease may experience complications, long-term abnormal blood sugar levels, unhealthy diet habits, and different environments; hence, it is challenging for observational studies to adjust for all confounders (2). Therefore, residual confounding or reverse causation bias in observational research may explain our disagreement. The present study used an MR design to confirm the causality pathway from T1DM to six high-frequency infections. As no heterogeneity was observed in the MR analysis of T1DM on IIs, sepsis, ALRIs, GUTIs in pregnancy, and UTIs, we selected the IVW-fixed analysis results as our primary results. As significant heterogeneity was observed in the MR analysis of T1DM on SSTIs, we selected the IVW-random analysis result as the primary result. We also conducted different MR methods as complementary and sensitivity analyses to confirm our results. Moreover, to strictly meet the criteria of an “IVs not linked to confounding factors” and “IVs influence outcomes only through T1DM,” we deleted all SNPs potentially associated with confounders and outcomes according to the trait in PhenoScanner V2.

Approximately 451 million people have diabetes mellitus globally, approximately 5 million deaths were attributed to diabetes mellitus (DM) in 2017, and the number of people with DM may increase to 693 million by 2045 (50). Recently, Wang et al. conducted an MR to explore the causal relationship between T2DM and five high-frequency infectious diseases, including UTIs in pregnancy, ALRIs, sepsis, and SSTIs. They stated that no causal relationships existed (51). The present MR study complemented and extended the abovementioned study by Wang et al. In this study, we not only confirmed that T1DM has no causality with those five high-frequency infectious diseases but also found that T1DM increased the occurrence of IIs due to genetic liability.

Early in 2007, Oikarinen et al. examined enteroviruses in small intestinal tissue of T1DM and suggested that most had a persistent enterovirus infection of the intestinal mucosa (52). A later case-control study confirmed that enterovirus RNA was found more frequently in patients with T1DM than in controls, and the enterovirus was considered to be associated with IIs (53).

The potential mechanism between T1DM and IIs may be explained by the following three pathways. First, many studies have reported dysbiosis of the intestinal microbiomes in patients with T1DM, which would promote inflammation in the gut (49, 54). Second, a review article suggested that a genetically deficient macrophage migration inhibitory factor, one of the mechanisms for the occurrence of T1DM, would induce the Th1 inflammatory cytokines and reduce regulatory T cells (Treg), thereby finally causing colitis (55). Third, celiac disease primarily affects the small intestine, causes weight loss, anemia, and intestinal mucosal damage, and has been found to be associated with T1DM (56, 57). Both celiac disease and T1DM are linked to high-risk human lymphocyte antigens (HLAs) (58). HLAs present antigens to antigen-responsive T cells, resulting in the destruction of the intestinal enterocyte (58).

Based on our positive findings, we provide some prevention strategies for infections in T1DM. First, hand hygiene: good hand hygiene is the most important step in preventing infections. Second, food hygiene: individuals with T1DM should ensure that their food is clean and thoroughly cooked. Third, immunization: individuals with T1DM should receive all recommended vaccinations, especially some vaccines to prevent intestinal infections, such as the rotavirus vaccine, enterohemorrhagic *E. coli* vaccine, and salmonella vaccine (59). Fourth, blood sugar control: individuals with T1DM should aim to maintain good glycemic control to reduce the risk of infections. Fifth, probiotics are live microorganisms that can help to promote a healthy gut microbiome and prevent intestinal infections. T1DM can take probiotic supplements. Sixth, follow a strict gluten-free diet when T1DM is complicated with celiac disease (60).

The MR study design, which reduces confounders and reverse causality which may influence the findings of epidemiological studies, is a key strength of this work. Additionally, we systematically explored the causalities between T1DM and six common infections. For all the selected IVs, F-statistics were more than 10. Furthermore, no pleiotropy was detected, confirming our results’ accuracy. After a causal association between T1DM and IIs was detected, we performed three different MVMR analyses (MVMR-IVW, LASSO, and MVMR-Robust) to assess the validity of the results by adjusting for BMI and HbA1c. LASSO and MVMR-Robust provided a reliable estimation even with high pleiotropy, and no heterogeneity was observed; the results obtained using the three methods were consistent, demonstrating their credibility. The datasets of exposure and outcomes were mainly composed of European populations; consequently, these findings were less likely to be influenced by population stratification, although it limits the applicability to other ethnic groups. Moreover, we presented a checklist based on the previous work by Woolf et al. (61) to improve our reporting quality, and most of the list items were attained. Since we could not obtain detailed information from the original research, it was challenging to evaluate the quality of the data sources (**Supplementary Table 9**). We only chose recent datasets provided by widely recognized consortiums to address this limitation. Furthermore, we cannot exclude the possibility that other potential confounders may have influenced our results. We excluded all SNPs associated with more than ten potential confounders possibly associated with the outcomes and performed three MVMR methods adjusting for BMI and HbA1c. However, we cannot rule out all the direct pathways between T1DM and IIs, as pleiotropy could not be controlled entirely. Since some previous studies showed some infectious diseases might cause T1DM, a bidirectional MR analysis should be performed to explore the association of infectious diseases with T1DM. However, none of the six infectious diseases GWAS datasets could supply sufficient SNPs (clumped at p< 5 × 10^−8^, kb=10,000, and r^2^<0.001) to perform MR and sensitivity analyses. Therefore, this was one limitation we could not improve and should be performed in future studies.

In conclusion, our study found no convincing evidence of a causal link between T1DM and sepsis, ALRIs, GUTIs in pregnancy, SSTIs, or UTIs in the European population. However, there was a genetic liability in T1DM for increased risk of contracting IIs. Larger epidemiological studies or metagenomic studies are needed to better understand the observed association between susceptibility of certain infectious diseases in patients with T1DM.

## Data availability statement

The datasets presented in this study can be found in online repositories. The names of the repository/repositories and accession number(s) can be found below: The datasets [T1DM, sepsis, ALRIs, GUTI susceptibility in pregnancy, SSTIs, UTIs, IIs] for this study can be found in the GWAS summary data [https://gwas.mrcieu.ac.uk/].

## Ethics statement

Ethical review and approval was not required for the study on human participants in accordance with the local legislation and institutional requirements. Written informed consent for participation was not required for this study in accordance with the national legislation and the institutional requirements.

## Author contributions

TX, HW, and X-HC contributed to the conception and design of the study. H-QL and QN organized the database. X-HC and QN performed the statistical analyses. X-HC and H-QL wrote the first draft of the manuscript. All authors contributed to the article and approved the submitted version.

## Glossary

**Table:**

Term	Definition
**Single nucleotide polymorphism (SNP)**	A variation at a single position in a DNA sequence that occurs when one nucleotide is replaced by another in at least 1% of the population. SNPs can result in different alleles at that position.
**Trait**	A phenotype of an SNP.
**Genome-wide association study (GWAS)**	A genetic study in which the phenotype is regressed for each genetic variant to correct the bias resulting from multiple testing.
**Genetic variant**	Any change in the DNA sequence that distinguishes one individual from another. This can include variations such as SNPs.
**Harmonization**	Some SNPs may have more than one possible allele. Harmonization is the process of formatting GWAS statistics to ensure that exposures and outcomes use the same allele as the effect allele; otherwise, the Mendelian randomization analysis results will be incorrect.
**Palindromic variants**	An SNP in which the same two nucleotides are found on both forward and reverse strands (e.g., A/T on the forward and T/A on the reverse). This can cause problems in harmonization because it is difficult to establish which is the minor allele in cases where both nucleotides have a similar frequency.
**Mendelian randomization (MR) analysis**	The statistical analysis used in a two-sample MR design, which estimates the cause association of the exposure on the outcome.
**Multivariable Mendelian randomization (MVMR)**	A statistical technique that uses genetic variants to investigate the causal relationships between multiple exposure variables and an outcome variable.
**Instruments**	Instruments were randomly assigned, associated with the exposure, and performed in MR analyses to infer the causal association between exposure and outcome.
**Linkage disequilibrium (LD)**	A non-random association between nearby genetic variants. In MR studies, LD can lead to biased results if not accounted for. Researchers adjust for LD or select independent variants as instruments to avoid this.
**Proxy variant**	Some SNPs of exposure cannot be extracted from the dataset of the outcome. We could use other SNPs as proxy variants highly correlated with the unavailable SNPs to perform the MR analysis.
**Genetic liability to the exposure**	Binary traits with many associated genetic variants have an underlying continuous genetic liability measured through GWASs. People’s genes can make them more or less likely to be affected by environmental factors that can harm them, like pollution or toxins. Some people may have genes that make them more sensitive to these things, while others may have genes that make them less sensitive.
**Pleiotropy**	Pleiotropy in MR occurs when the genetic instruments used to estimate the causal effect of an exposure on an outcome also directly affect the outcome through other pathways.
**Heterogeneity**	Heterogeneity indicates a violation of the exclusion restriction assumption in MR (validity), which can lead to biased effect estimates.
**Cochran’s Q statistic**	A statistical test is used to determine whether there is significant heterogeneity (variation) in the effect sizes of multiple studies included in a meta-analysis.
**Population stratification**	The uneven distribution of genetic variants between different subpopulations can bias associations between exposures and outcomes in genetic studies.
