# Supplementary material for: Causal relationship between type 1 diabetes mellitus and six high-frequency infectious diseases: A two-sample mendelian randomization study
Source: Front Endocrinol (Lausanne). 2023 Mar 31;14:1135726. doi: 10.3389/fendo.2023.1135726 (PMC10102543; doi:10.3389/fendo.2023.1135726)
Supplement: Supplementary file 1 [file DataSheet_1.docx]

Supplementary Material

Causal Relationship Between Type 1 Diabetes Mellitus and Six High-Frequency Infectious Diseases: A Two-Sample Mendelian Randomization Study

Xiao-Hong Chen, Hong-Qiong Liu, Qiong Nie, Han Wang*, Tao Xiang*

* Correspondence:

Tao Xiang: xt1142752929@163.com

Han Wang: wanghan@swjtu.edu.cn

# Supplementary Tables

**Table S1** Detailed information on the genetic association datasets of confounders.

| **Exposure** | **GWAS ID** | **Consortium** | **Sample Size** | **Year** |  | **Population** |
| --- | --- | --- | --- | --- | --- | --- |
| BMI | ieu-b-4816 | Within family GWAS consortium | 99,998 | 2022 |  | European |
| HbA1c | ieu-b-4842 | Within family GWAS consortium | 45,734 | 2022 |  | European |

**Abbreviations:** BMI, body mass index; HbA1c, glycated hemoglobin

**Table S2** Detailed information about 44 SNPs of type 1 diabetes mellitus.

| SNP | effect_allele | other_allele | β | SE | p-value |
| --- | --- | --- | --- | --- | --- |
| rs6679677 | A | C | 0.6527 | 0.0346 | 3.42E-79 |
| rs10911399 | G | A | -0.3707 | 0.064 | 6.75E-09 |
| rs2269247 | T | C | 0.1709 | 0.0295 | 7.28E-09 |
| rs1869449 | A | G | 0.1769 | 0.0269 | 4.55E-11 |
| rs10865468 | C | G | -0.1624 | 0.0277 | 4.66E-09 |
| rs11571297 | C | T | -0.1964 | 0.0237 | 1.11E-16 |
| rs10183097 | C | T | 0.2053 | 0.0322 | 1.82E-10 |
| rs192324744 | G | T | 0.562 | 0.0875 | 1.36E-10 |
| rs6719660 | G | A | 0.2918 | 0.0524 | 2.52E-08 |
| rs2111485 | G | A | 0.1577 | 0.0248 | 1.89E-10 |
| rs17863786 | G | A | 0.4144 | 0.0628 | 4.26E-11 |
| rs1027769 | T | G | -0.9962 | 0.1588 | 3.52E-10 |
| rs62410259 | A | G | -0.3796 | 0.0533 | 1.02E-12 |
| rs34954 | A | C | -0.4912 | 0.0863 | 1.25E-08 |
| rs13182737 | A | G | 0.1465 | 0.0259 | 1.49E-08 |
| rs9273363 | A | C | 1.2786 | 0.0334 | 1E-200 |
| rs9296062 | C | G | 0.6913 | 0.054 | 1.37E-37 |
| rs6909461 | C | A | -0.314 | 0.0332 | 3.06E-21 |
| rs506770 | C | G | 1.0048 | 0.0426 | 3.3266E-123 |
| rs185774696 | T | C | 0.6489 | 0.0418 | 2.66E-54 |
| rs9468618 | T | C | -0.3009 | 0.0489 | 7.53E-10 |
| rs206763 | A | G | 0.6792 | 0.0779 | 2.93E-18 |
| rs2144013 | G | A | 0.2234 | 0.0317 | 1.76E-12 |
| rs34296259 | A | T | 0.6637 | 0.1171 | 1.43E-08 |
| rs10760335 | G | A | 0.1357 | 0.0243 | 2.43E-08 |
| rs77523242 | C | T | -0.3705 | 0.0635 | 5.42E-09 |
| rs12722495 | C | T | -0.3145 | 0.0408 | 1.27E-14 |
| rs689 | T | A | 0.7004 | 0.0354 | 2.30E-87 |
| rs10830227 | A | G | 0.1582 | 0.0233 | 1.02E-11 |
| rs79075295 | A | G | -0.4192 | 0.0621 | 1.46E-11 |
| rs1131017 | G | C | -0.2461 | 0.0238 | 4.24E-25 |
| rs59680223 | T | C | 0.6421 | 0.1032 | 5.00E-10 |
| rs10774624 | A | G | -0.2556 | 0.0244 | 1.34E-25 |
| rs2071647 | A | T | 0.1526 | 0.0258 | 3.30E-09 |
| rs194749 | C | T | -0.1638 | 0.0281 | 5.37E-09 |
| rs201417739 | C | A | -0.416 | 0.0663 | 3.41E-10 |
| rs55996894 | C | G | -0.1785 | 0.0323 | 3.13E-08 |
| rs17125653 | A | T | 0.2355 | 0.0402 | 4.75E-09 |
| rs4566101 | C | T | 0.1755 | 0.0255 | 6.23E-12 |
| rs741172 | T | C | -0.2034 | 0.0258 | 3.11E-15 |
| rs231971 | G | A | 0.2411 | 0.0399 | 1.55E-09 |
| rs8056814 | A | G | 0.2641 | 0.0415 | 1.99E-10 |
| rs34536443 | C | G | -0.4139 | 0.0665 | 4.84E-10 |

SNP, single nucleotide polymorphism; SE, standard error.

**Table S3** Detailed information about SNPs of type 1 diabetes mellitus on intestinal infections.

| **SNP** | **effect_allele exposure** | **other_alleleexposure** | **eaf.exposure** | **beta.exposure** | **se.exposure** | **pval.exposure** | **beta.IIs** | **se.IIs** | **pval.IIs** |
| --- | --- | --- | --- | --- | --- | --- | --- | --- | --- |
| rs10183097 | C | T | 0.1362 | 0.2053 | 0.0322 | 1.82E-10 | -0.0052 | 0.0316 | 0.868 |
| rs1027769 | T | G | 0.9966 | -0.9962 | 0.1588 | 3.52E-10 | 0.0538 | 0.0736 | 0.4649 |
| rs10760335 | G | A | 0.3205 | 0.1357 | 0.0243 | 2.43E-08 | 0.0197 | 0.0245 | 0.4209 |
| rs10774624 | A | G | 0.5037 | -0.2556 | 0.0244 | 1.34E-25 | -0.0365 | 0.0232 | 0.1156 |
| rs10865468 | C | G | 0.2527 | -0.1624 | 0.0277 | 4.66E-09 | 0.0066 | 0.0244 | 0.786799 |
| rs10911399 | G | A | 0.0455 | -0.3707 | 0.064 | 6.75E-09 | -0.0545 | 0.0695 | 0.4334 |
| rs11571297 | C | T | 0.4844 | -0.1964 | 0.0237 | 1.11E-16 | 0.0116 | 0.0234 | 0.6205 |
| rs12722495 | C | T | 0.1122 | -0.3145 | 0.0408 | 1.27E-14 | -0.0355 | 0.0457 | 0.437 |
| rs13182737 | A | G | 0.2553 | 0.1465 | 0.0259 | 1.49E-08 | 0.0283 | 0.0249 | 0.2556 |
| rs17125653 | A | T | 0.077 | 0.2355 | 0.0402 | 4.75E-09 | -0.0311 | 0.0334 | 0.3507 |
| rs1869449 | A | G | 0.2967 | 0.1769 | 0.0269 | 4.55E-11 | -0.0069 | 0.0255 | 0.7854 |
| rs192324744 | G | T | 0.0131 | 0.562 | 0.0875 | 1.36E-10 | -0.0048 | 0.0938 | 0.9594 |
| rs194749 | C | T | 0.2455 | -0.1638 | 0.0281 | 5.37E-09 | -0.0133 | 0.0252 | 0.597601 |
| rs202520 | G | A | 0.7222 | -0.1573 | 0.0256 | 7.97E-10 | -0.0087 | 0.024 | 0.717399 |
| rs206763 | A | G | 0.0202 | 0.6792 | 0.0779 | 2.93E-18 | -0.1046 | 0.0932 | 0.2618 |
| rs2071647 | A | T | 0.2755 | 0.1526 | 0.0258 | 3.30E-09 | 0.002 | 0.0257 | 0.9382 |
| rs2144013 | G | A | 0.2093 | 0.2234 | 0.0317 | 1.76E-12 | 0.0411 | 0.0287 | 0.1529 |
| rs2269247 | T | C | 0.1804 | 0.1709 | 0.0295 | 7.28E-09 | -0.0253 | 0.0245 | 0.3025 |
| rs231971 | G | A | 0.1026 | 0.2411 | 0.0399 | 1.55E-09 | 0.0787 | 0.0583 | 0.1774 |
| rs34296259 | A | T | 0.0082 | 0.6637 | 0.1171 | 1.43E-08 | 0.0747 | 0.0507 | 0.1408 |
| rs34536443 | C | G | 0.0515 | -0.4139 | 0.0665 | 4.84E-10 | -0.0115 | 0.0674 | 0.8645 |
| rs34954 | A | C | 0.0443 | -0.4912 | 0.0863 | 1.25E-08 | 0.016 | 0.0427 | 0.7083 |
| rs4566101 | C | T | 0.2697 | 0.1755 | 0.0255 | 6.23E-12 | 0.0088 | 0.0251 | 0.7274 |
| rs506770 | C | G | 0.768 | 1.0048 | 0.0426 | 3.33E-123 | 0.0592 | 0.028 | 0.03473 |
| rs55996894 | C | G | 0.204 | -0.1785 | 0.0323 | 3.13E-08 | -0.0248 | 0.0319 | 0.4385 |
| rs59680223 | T | C | 0.0081 | 0.6421 | 0.1032 | 5.00E-10 | 0.055 | 0.0891 | 0.5369 |
| rs62410259 | A | G | 0.077 | -0.3796 | 0.0533 | 1.02E-12 | -0.0242 | 0.0419 | 0.5633 |
| rs6719660 | G | A | 0.9354 | 0.2918 | 0.0524 | 2.52E-08 | 0.0588 | 0.0588 | 0.3171 |
| rs689 | T | A | 0.7109 | 0.7004 | 0.0354 | 2.30E-87 | 0.0636 | 0.0283 | 0.02459 |
| rs6909461 | C | A | 0.2529 | -0.314 | 0.0332 | 3.06E-21 | 0.0351 | 0.0292 | 0.2292 |
| rs741172 | T | C | 0.3205 | -0.2034 | 0.0258 | 3.11E-15 | 0.0109 | 0.025 | 0.6638 |
| rs8056814 | A | G | 0.0793 | 0.2641 | 0.0415 | 1.99E-10 | 0.0217 | 0.0409 | 0.5949 |
| rs9296062 | C | G | 0.0509 | 0.6913 | 0.054 | 1.37E-37 | 0.0782 | 0.0432 | 0.070201 |
| rs9468618 | T | C | 0.0975 | -0.3009 | 0.0489 | 7.53E-10 | -0.1775 | 0.0499 | 0.00038 |

SNP, single nucleotide polymorphism; se, standard error; pval, p-value; IIs, intestinal infections.

**Table S4** Detailed information about SNPs of type 1 diabetes mellitus on sepsis.

| **SNP** | **effect_allele exposure** | **other_alleleexposure** | **eaf.exposure** | **beta.exposure** | **se.exposure** | **pval.exposure** | **beta.sepsis** | **se.sepsis** | **pval.sepsis** |
| --- | --- | --- | --- | --- | --- | --- | --- | --- | --- |
| rs10183097 | C | T | 0.1362 | 0.2053 | 0.0322 | 1.82E-10 | 0.011984 | 0.0198 | 0.545001 |
| rs10760335 | G | A | 0.3205 | 0.1357 | 0.0243 | 2.43E-08 | -0.01964 | 0.01461 | 0.178852 |
| rs10774624 | A | G | 0.5037 | -0.2556 | 0.0244 | 1.34E-25 | 0.012355 | 0.013818 | 0.371226 |
| rs10865468 | C | G | 0.2527 | -0.1624 | 0.0277 | 4.66E-09 | -0.00408 | 0.015818 | 0.796663 |
| rs10911399 | G | A | 0.0455 | -0.3707 | 0.064 | 6.75E-09 | 0.021012 | 0.040443 | 0.603372 |
| rs11571297 | C | T | 0.4844 | -0.1964 | 0.0237 | 1.11E-16 | -0.00891 | 0.013748 | 0.516912 |
| rs12722495 | C | T | 0.1122 | -0.3145 | 0.0408 | 1.27E-14 | 0.02034 | 0.021895 | 0.352904 |
| rs13182737 | A | G | 0.2553 | 0.1465 | 0.0259 | 1.49E-08 | 0.033484 | 0.01564 | 0.032282 |
| rs17125653 | A | T | 0.077 | 0.2355 | 0.0402 | 4.75E-09 | -0.00922 | 0.02519 | 0.714279 |
| rs17863786 | G | A | 0.0293 | 0.4144 | 0.0628 | 4.26E-11 | 0.080343 | 0.043528 | 0.064926 |
| rs185774696 | T | C | 0.1772 | 0.6489 | 0.0418 | 2.66E-54 | 0.007484 | 0.017422 | 0.66752 |
| rs1869449 | A | G | 0.2967 | 0.1769 | 0.0269 | 4.55E-11 | 0.010338 | 0.014953 | 0.489361 |
| rs192324744 | G | T | 0.0131 | 0.562 | 0.0875 | 1.36E-10 | 0.057321 | 0.053549 | 0.284421 |
| rs194749 | C | T | 0.2455 | -0.1638 | 0.0281 | 5.37E-09 | 0.035553 | 0.015965 | 0.02595 |
| rs201417739 | C | A | 0.075 | -0.416 | 0.0663 | 3.41E-10 | 0.065017 | 0.045447 | 0.152546 |
| rs202520 | G | A | 0.7222 | -0.1573 | 0.0256 | 7.97E-10 | -0.01025 | 0.015224 | 0.500879 |
| rs206763 | A | G | 0.0202 | 0.6792 | 0.0779 | 2.93E-18 | 0.07792 | 0.951369 | 0.934724 |
| rs206763 | A | G | 0.0202 | 0.6792 | 0.0779 | 2.93E-18 | -0.02147 | 0.05055 | 0.670987 |
| rs2071647 | A | T | 0.2755 | 0.1526 | 0.0258 | 3.30E-09 | 0.026077 | 0.015368 | 0.089737 |
| rs2144013 | G | A | 0.2093 | 0.2234 | 0.0317 | 1.76E-12 | -0.03276 | 0.016901 | 0.052588 |
| rs2269247 | T | C | 0.1804 | 0.1709 | 0.0295 | 7.28E-09 | 0.00561 | 0.017728 | 0.751652 |
| rs231971 | G | A | 0.1026 | 0.2411 | 0.0399 | 1.55E-09 | -0.03684 | 0.025135 | 0.142693 |
| rs34296259 | A | T | 0.0082 | 0.6637 | 0.1171 | 1.43E-08 | -0.09163 | 0.063127 | 0.146636 |
| rs34536443 | C | G | 0.0515 | -0.4139 | 0.0665 | 4.84E-10 | -0.04584 | 0.033712 | 0.173925 |
| rs34954 | A | C | 0.0443 | -0.4912 | 0.0863 | 1.25E-08 | 0.032872 | 0.024459 | 0.178953 |
| rs34954 | A | C | 0.0443 | -0.4912 | 0.0863 | 1.25E-08 | 0.073729 | 0.051033 | 0.148528 |
| rs4566101 | C | T | 0.2697 | 0.1755 | 0.0255 | 6.23E-12 | -0.0152 | 0.015432 | 0.324638 |
| rs506770 | C | G | 0.768 | 1.0048 | 0.0426 | 3.33E-123 | -0.00108 | 0.015994 | 0.946394 |
| rs55996894 | C | G | 0.204 | -0.1785 | 0.0323 | 3.13E-08 | 0.001387 | 0.017938 | 0.93838 |
| rs59680223 | T | C | 0.0081 | 0.6421 | 0.1032 | 5.00E-10 | -0.10419 | 0.077342 | 0.177926 |
| rs62410259 | A | G | 0.077 | -0.3796 | 0.0533 | 1.02E-12 | -0.04408 | 0.026031 | 0.090375 |
| rs6719660 | G | A | 0.9354 | 0.2918 | 0.0524 | 2.52E-08 | 0.027553 | 0.030035 | 0.358958 |
| rs689 | T | A | 0.7109 | 0.7004 | 0.0354 | 2.30E-87 | -0.02998 | 0.015157 | 0.04794 |
| rs6909461 | C | A | 0.2529 | -0.314 | 0.0332 | 3.06E-21 | -0.00104 | 0.016383 | 0.949393 |
| rs741172 | T | C | 0.3205 | -0.2034 | 0.0258 | 3.11E-15 | -0.00763 | 0.014643 | 0.602407 |
| rs77523242 | C | T | 0.0623 | -0.3705 | 0.0635 | 5.42E-09 | -0.02469 | 0.029625 | 0.404555 |
| rs79075295 | A | G | 0.0921 | -0.4192 | 0.0621 | 1.46E-11 | -0.02322 | 0.039026 | 0.551911 |
| rs8056814 | A | G | 0.0793 | 0.2641 | 0.0415 | 1.99E-10 | -0.01992 | 0.024685 | 0.419591 |
| rs9296062 | C | G | 0.0509 | 0.6913 | 0.054 | 1.37E-37 | 0.014946 | 0.032847 | 0.649084 |

SNP, single nucleotide polymorphism; se, standard error; pval, p-value.

**Table S5** Detailed information about SNPs of type 1 diabetes mellitus on acute lower respiratory infections.

| **SNP** | **effect_allele exposure** | **other_alleleexposure** | **eaf.exposure** | **beta.exposure** | **se.exposure** | **pval. exposure** | **beta. ALRIs** | **se. ALRIs** | **pval. ALRIs** |
| --- | --- | --- | --- | --- | --- | --- | --- | --- | --- |
| rs10183097 | C | T | 0.1362 | 0.2053 | 0.0322 | 1.82E-10 | -0.0086 | 0.0205 | 0.6747 |
| rs1027769 | T | G | 0.9966 | -0.9962 | 0.1588 | 3.52E-10 | -0.1034 | 0.0485 | 0.03296 |
| rs10760335 | G | A | 0.3205 | 0.1357 | 0.0243 | 2.43E-08 | -0.0143 | 0.0158 | 0.3664 |
| rs10774624 | A | G | 0.5037 | -0.2556 | 0.0244 | 1.34E-25 | -0.0252 | 0.015 | 0.092421 |
| rs10865468 | C | G | 0.2527 | -0.1624 | 0.0277 | 4.66E-09 | -0.0107 | 0.0158 | 0.500299 |
| rs10911399 | G | A | 0.0455 | -0.3707 | 0.064 | 6.75E-09 | -0.0413 | 0.0451 | 0.3601 |
| rs11571297 | C | T | 0.4844 | -0.1964 | 0.0237 | 1.11E-16 | 0.0015 | 0.0151 | 0.9216 |
| rs12722495 | C | T | 0.1122 | -0.3145 | 0.0408 | 1.27E-14 | -0.0393 | 0.0297 | 0.1848 |
| rs13182737 | A | G | 0.2553 | 0.1465 | 0.0259 | 1.49E-08 | -0.017 | 0.0161 | 0.2919 |
| rs17125653 | A | T | 0.077 | 0.2355 | 0.0402 | 4.75E-09 | 0.0221 | 0.0216 | 0.3073 |
| rs1869449 | A | G | 0.2967 | 0.1769 | 0.0269 | 4.55E-11 | 0.0071 | 0.0165 | 0.664899 |
| rs192324744 | G | T | 0.0131 | 0.562 | 0.0875 | 1.36E-10 | 0.0924 | 0.0598 | 0.1222 |
| rs194749 | C | T | 0.2455 | -0.1638 | 0.0281 | 5.37E-09 | -0.0095 | 0.0163 | 0.5592 |
| rs202520 | G | A | 0.7222 | -0.1573 | 0.0256 | 7.97E-10 | 0.0236 | 0.0155 | 0.1282 |
| rs206763 | A | G | 0.0202 | 0.6792 | 0.0779 | 2.93E-18 | 0.0166 | 0.0592 | 0.779301 |
| rs2071647 | A | T | 0.2755 | 0.1526 | 0.0258 | 3.30E-09 | 0.0336 | 0.0166 | 0.04335 |
| rs2144013 | G | A | 0.2093 | 0.2234 | 0.0317 | 1.76E-12 | -0.0064 | 0.0186 | 0.730801 |
| rs2269247 | T | C | 0.1804 | 0.1709 | 0.0295 | 7.28E-09 | 0.0033 | 0.0159 | 0.8367 |
| rs231971 | G | A | 0.1026 | 0.2411 | 0.0399 | 1.55E-09 | -0.0185 | 0.0381 | 0.6271 |
| rs34296259 | A | T | 0.0082 | 0.6637 | 0.1171 | 1.43E-08 | 0.0253 | 0.0329 | 0.4418 |
| rs34536443 | C | G | 0.0515 | -0.4139 | 0.0665 | 4.84E-10 | 0.0431 | 0.0429 | 0.3155 |
| rs34954 | A | C | 0.0443 | -0.4912 | 0.0863 | 1.25E-08 | -0.0009 | 0.0275 | 0.9729 |
| rs4566101 | C | T | 0.2697 | 0.1755 | 0.0255 | 6.23E-12 | 0.0095 | 0.0163 | 0.5582 |
| rs506770 | C | G | 0.768 | 1.0048 | 0.0426 | 3.33E-123 | 0.0065 | 0.0181 | 0.7185 |
| rs55996894 | C | G | 0.204 | -0.1785 | 0.0323 | 3.13E-08 | -0.0065 | 0.0206 | 0.7516 |
| rs59680223 | T | C | 0.0081 | 0.6421 | 0.1032 | 5.00E-10 | -0.0642 | 0.0581 | 0.2695 |
| rs62410259 | A | G | 0.077 | -0.3796 | 0.0533 | 1.02E-12 | 0.0038 | 0.027 | 0.8871 |
| rs6719660 | G | A | 0.9354 | 0.2918 | 0.0524 | 2.52E-08 | 0.0397 | 0.0378 | 0.2929 |
| rs689 | T | A | 0.7109 | 0.7004 | 0.0354 | 2.30E-87 | 6.00E-04 | 0.0182 | 0.9715 |
| rs6909461 | C | A | 0.2529 | -0.314 | 0.0332 | 3.06E-21 | 0.0019 | 0.019 | 0.9206 |
| rs741172 | T | C | 0.3205 | -0.2034 | 0.0258 | 3.11E-15 | -0.0139 | 0.0162 | 0.3911 |
| rs8056814 | A | G | 0.0793 | 0.2641 | 0.0415 | 1.99E-10 | -0.0233 | 0.0266 | 0.3821 |
| rs9296062 | C | G | 0.0509 | 0.6913 | 0.054 | 1.37E-37 | 0.0103 | 0.0282 | 0.7147 |
| rs9468618 | T | C | 0.0975 | -0.3009 | 0.0489 | 7.53E-10 | -0.0421 | 0.032 | 0.1883 |

SNP, single nucleotide polymorphism; se, standard error; pval, p-value; ALRIs, acute lower respiratory infections.

**Table S6** Detailed information about SNPs of type 1 diabetes mellitus on genitourinary tract infections in pregnancy.

| **SNP** | **effect_allele exposure** | **other_alleleexposure** | **eaf.exposure** | **beta.exposure** | **se.exposure** | **pval.exposure** | **beta.GUTIs in pregnancy** | **se. GUTIs in pregnancy** | **pval. GUTIs in pregnancy** |
| --- | --- | --- | --- | --- | --- | --- | --- | --- | --- |
| rs10183097 | C | T | 0.1362 | 0.2053 | 0.0322 | 1.82E-10 | -0.0186 | 0.0534 | 0.728201 |
| rs1027769 | T | G | 0.9966 | -0.9962 | 0.1588 | 3.52E-10 | -0.0468 | 0.126 | 0.710101 |
| rs10760335 | G | A | 0.3205 | 0.1357 | 0.0243 | 2.43E-08 | -0.0178 | 0.0413 | 0.6659 |
| rs10774624 | A | G | 0.5037 | -0.2556 | 0.0244 | 1.34E-25 | 0.0548 | 0.0391 | 0.1604 |
| rs10865468 | C | G | 0.2527 | -0.1624 | 0.0277 | 4.66E-09 | 0.027 | 0.0413 | 0.5133 |
| rs10911399 | G | A | 0.0455 | -0.3707 | 0.064 | 6.75E-09 | -0.0234 | 0.1171 | 0.8417 |
| rs11571297 | C | T | 0.4844 | -0.1964 | 0.0237 | 1.11E-16 | 0.034 | 0.0395 | 0.3891 |
| rs12722495 | C | T | 0.1122 | -0.3145 | 0.0408 | 1.27E-14 | -0.0424 | 0.0773 | 0.5833 |
| rs13182737 | A | G | 0.2553 | 0.1465 | 0.0259 | 1.49E-08 | -0.0125 | 0.042 | 0.7666 |
| rs17125653 | A | T | 0.077 | 0.2355 | 0.0402 | 4.75E-09 | -0.0299 | 0.0562 | 0.5943 |
| rs1869449 | A | G | 0.2967 | 0.1769 | 0.0269 | 4.55E-11 | 0.0519 | 0.043 | 0.2274 |
| rs192324744 | G | T | 0.0131 | 0.562 | 0.0875 | 1.36E-10 | -0.1808 | 0.1562 | 0.247 |
| rs194749 | C | T | 0.2455 | -0.1638 | 0.0281 | 5.37E-09 | 0.0338 | 0.0425 | 0.427 |
| rs202520 | G | A | 0.7222 | -0.1573 | 0.0256 | 7.97E-10 | 0.045 | 0.0404 | 0.2657 |
| rs206763 | A | G | 0.0202 | 0.6792 | 0.0779 | 2.93E-18 | 0.0764 | 0.1537 | 0.6193 |
| rs2071647 | A | T | 0.2755 | 0.1526 | 0.0258 | 3.30E-09 | 0.0122 | 0.0436 | 0.7799 |
| rs2144013 | G | A | 0.2093 | 0.2234 | 0.0317 | 1.76E-12 | -0.0497 | 0.0486 | 0.3062 |
| rs2269247 | T | C | 0.1804 | 0.1709 | 0.0295 | 7.28E-09 | 0.0074 | 0.0414 | 0.8573 |
| rs231971 | G | A | 0.1026 | 0.2411 | 0.0399 | 1.55E-09 | 0.0303 | 0.1001 | 0.7619 |
| rs34296259 | A | T | 0.0082 | 0.6637 | 0.1171 | 1.43E-08 | 0.0716 | 0.0861 | 0.4056 |
| rs34536443 | C | G | 0.0515 | -0.4139 | 0.0665 | 4.84E-10 | -0.0779 | 0.1122 | 0.4872 |
| rs34954 | A | C | 0.0443 | -0.4912 | 0.0863 | 1.25E-08 | -0.0174 | 0.0719 | 0.8088 |
| rs4566101 | C | T | 0.2697 | 0.1755 | 0.0255 | 6.23E-12 | -0.0078 | 0.0424 | 0.8545 |
| rs506770 | C | G | 0.768 | 1.0048 | 0.0426 | 3.33E-123 | 0.0149 | 0.0474 | 0.752899 |
| rs55996894 | C | G | 0.204 | -0.1785 | 0.0323 | 3.13E-08 | 2.00E-04 | 0.054 | 0.9973 |
| rs59680223 | T | C | 0.0081 | 0.6421 | 0.1032 | 5.00E-10 | -0.0453 | 0.1524 | 0.7662 |
| rs62410259 | A | G | 0.077 | -0.3796 | 0.0533 | 1.02E-12 | 0.0148 | 0.0706 | 0.834 |
| rs6719660 | G | A | 0.9354 | 0.2918 | 0.0524 | 2.52E-08 | 0.1363 | 0.0986 | 0.167 |
| rs689 | T | A | 0.7109 | 0.7004 | 0.0354 | 2.30E-87 | -0.0387 | 0.0475 | 0.415 |
| rs6909461 | C | A | 0.2529 | -0.314 | 0.0332 | 3.06E-21 | 0.0929 | 0.0495 | 0.0606694 |
| rs741172 | T | C | 0.3205 | -0.2034 | 0.0258 | 3.11E-15 | -0.0374 | 0.0421 | 0.3752 |
| rs8056814 | A | G | 0.0793 | 0.2641 | 0.0415 | 1.99E-10 | 0.0128 | 0.0689 | 0.8527 |
| rs9296062 | C | G | 0.0509 | 0.6913 | 0.054 | 1.37E-37 | -0.0205 | 0.0731 | 0.7792 |
| rs9468618 | T | C | 0.0975 | -0.3009 | 0.0489 | 7.53E-10 | -0.2946 | 0.0839 | 0.000448497 |

SNP, single nucleotide polymorphism; se, standard error; pval, p-value; GUTIs, genitourinary tract infections.

**Table S7** Detailed information about SNPs of type 1 diabetes mellitus on infections of the skin and subcutaneous tissue.

| **SNP** | **effect_allele exposure** | **other_alleleexposure** | **eaf.exposure** | **beta.exposure** | **se.exposure** | **pval.exposure** | **beta.SSTIs** | **se. SSTIs** | **pval. SSTIs** |
| --- | --- | --- | --- | --- | --- | --- | --- | --- | --- |
| rs10183097 | C | T | 0.1362 | 0.2053 | 0.0322 | 1.82E-10 | 0.0281 | 0.0205 | 0.1701 |
| rs1027769 | T | G | 0.9966 | -0.9962 | 0.1588 | 3.52E-10 | 0.0798 | 0.0487 | 0.1013 |
| rs10760335 | G | A | 0.3205 | 0.1357 | 0.0243 | 2.43E-08 | -0.0023 | 0.0159 | 0.8848 |
| rs10774624 | A | G | 0.5037 | -0.2556 | 0.0244 | 1.34E-25 | -0.0283 | 0.0151 | 0.0600606 |
| rs10865468 | C | G | 0.2527 | -0.1624 | 0.0277 | 4.66E-09 | -0.0044 | 0.0159 | 0.7822 |
| rs10911399 | G | A | 0.0455 | -0.3707 | 0.064 | 6.75E-09 | 0.0671 | 0.0451 | 0.137 |
| rs11571297 | C | T | 0.4844 | -0.1964 | 0.0237 | 1.11E-16 | -0.0097 | 0.0152 | 0.5231 |
| rs12722495 | C | T | 0.1122 | -0.3145 | 0.0408 | 1.27E-14 | 0.0149 | 0.0299 | 0.6181 |
| rs13182737 | A | G | 0.2553 | 0.1465 | 0.0259 | 1.49E-08 | -0.001 | 0.0162 | 0.9507 |
| rs17125653 | A | T | 0.077 | 0.2355 | 0.0402 | 4.75E-09 | 0.059 | 0.0217 | 0.00652199 |
| rs1869449 | A | G | 0.2967 | 0.1769 | 0.0269 | 4.55E-11 | 0.0203 | 0.0165 | 0.2204 |
| rs192324744 | G | T | 0.0131 | 0.562 | 0.0875 | 1.36E-10 | 0.0583 | 0.0604 | 0.3342 |
| rs194749 | C | T | 0.2455 | -0.1638 | 0.0281 | 5.37E-09 | 3.00E-04 | 0.0164 | 0.9843 |
| rs202520 | G | A | 0.7222 | -0.1573 | 0.0256 | 7.97E-10 | -0.0063 | 0.0156 | 0.6856 |
| rs206763 | A | G | 0.0202 | 0.6792 | 0.0779 | 2.93E-18 | -0.0158 | 0.0594 | 0.7905 |
| rs2071647 | A | T | 0.2755 | 0.1526 | 0.0258 | 3.30E-09 | -0.0377 | 0.0167 | 0.0238798 |
| rs2144013 | G | A | 0.2093 | 0.2234 | 0.0317 | 1.76E-12 | -0.0242 | 0.0187 | 0.195 |
| rs2269247 | T | C | 0.1804 | 0.1709 | 0.0295 | 7.28E-09 | 0.0152 | 0.016 | 0.3427 |
| rs231971 | G | A | 0.1026 | 0.2411 | 0.0399 | 1.55E-09 | -0.0552 | 0.0382 | 0.1486 |
| rs34296259 | A | T | 0.0082 | 0.6637 | 0.1171 | 1.43E-08 | -0.0478 | 0.0329 | 0.1466 |
| rs34536443 | C | G | 0.0515 | -0.4139 | 0.0665 | 4.84E-10 | -0.0145 | 0.0431 | 0.737099 |
| rs34954 | A | C | 0.0443 | -0.4912 | 0.0863 | 1.25E-08 | -0.0073 | 0.0277 | 0.790999 |
| rs4566101 | C | T | 0.2697 | 0.1755 | 0.0255 | 6.23E-12 | -0.002 | 0.0163 | 0.9045 |
| rs506770 | C | G | 0.768 | 1.0048 | 0.0426 | 3.33E-123 | 0.0244 | 0.0182 | 0.1804 |
| rs55996894 | C | G | 0.204 | -0.1785 | 0.0323 | 3.13E-08 | -0.0553 | 0.0207 | 0.00766108 |
| rs59680223 | T | C | 0.0081 | 0.6421 | 0.1032 | 5.00E-10 | -0.0275 | 0.0581 | 0.636499 |
| rs62410259 | A | G | 0.077 | -0.3796 | 0.0533 | 1.02E-12 | 0.0132 | 0.0272 | 0.626599 |
| rs6719660 | G | A | 0.9354 | 0.2918 | 0.0524 | 2.52E-08 | 0.0101 | 0.0378 | 0.7899 |
| rs689 | T | A | 0.7109 | 0.7004 | 0.0354 | 2.30E-87 | -0.0046 | 0.0183 | 0.8004 |
| rs6909461 | C | A | 0.2529 | -0.314 | 0.0332 | 3.06E-21 | 0.0435 | 0.019 | 0.0223599 |
| rs741172 | T | C | 0.3205 | -0.2034 | 0.0258 | 3.11E-15 | -0.0037 | 0.0162 | 0.8206 |
| rs8056814 | A | G | 0.0793 | 0.2641 | 0.0415 | 1.99E-10 | -0.0129 | 0.0267 | 0.627899 |
| rs9296062 | C | G | 0.0509 | 0.6913 | 0.054 | 1.37E-37 | -9.00E-04 | 0.0282 | 0.9736 |
| rs9468618 | T | C | 0.0975 | -0.3009 | 0.0489 | 7.53E-10 | -0.0077 | 0.0321 | 0.8115 |

SNP, single nucleotide polymorphism; se, standard error; pval, p-value; SSTIs, infections of the skin and subcutaneous tissue.

**Table S8**  Detailed information about SNPs of type 1 diabetes mellitus on urinary tract infections.

| **SNP** | **effect_allele exposure** | **other_alleleexposure** | **eaf.exposure** | **beta.exposure** | **se.exposure** | **pval.exposure** | **beta.UTIs** | **se. UTIs** | **pval. UTIs** |
| --- | --- | --- | --- | --- | --- | --- | --- | --- | --- |
| rs10183097 | C | T | 0.1362 | 0.2053 | 0.0322 | 1.82E-10 | 1.39E-05 | 0.000324318 | 0.97 |
| rs10760335 | G | A | 0.3205 | 0.1357 | 0.0243 | 2.43E-08 | 0.000131871 | 0.000239641 | 0.58 |
| rs10774624 | A | G | 0.5037 | -0.2556 | 0.0244 | 1.34E-25 | -0.000360477 | 0.000226379 | 0.11 |
| rs10865468 | C | G | 0.2527 | -0.1624 | 0.0277 | 4.66E-09 | -0.000234012 | 0.000259533 | 0.37 |
| rs11571297 | C | T | 0.4844 | -0.1964 | 0.0237 | 1.11E-16 | 0.000340393 | 0.000225353 | 0.13 |
| rs12722495 | C | T | 0.1122 | -0.3145 | 0.0408 | 1.27E-14 | -0.000807514 | 0.000358986 | 0.0239999 |
| rs13182737 | A | G | 0.2553 | 0.1465 | 0.0259 | 1.49E-08 | 0.000277156 | 0.0002563 | 0.28 |
| rs17125653 | A | T | 0.077 | 0.2355 | 0.0402 | 4.75E-09 | -0.000367881 | 0.000414158 | 0.37 |
| rs185774696 | T | C | 0.1772 | 0.6489 | 0.0418 | 2.66E-54 | 0.000238707 | 0.000285889 | 0.4 |
| rs1869449 | A | G | 0.2967 | 0.1769 | 0.0269 | 4.55E-11 | 0.000328667 | 0.000245352 | 0.18 |
| rs194749 | C | T | 0.2455 | -0.1638 | 0.0281 | 5.37E-09 | 0.000242795 | 0.000262064 | 0.35 |
| rs202520 | G | A | 0.7222 | -0.1573 | 0.0256 | 7.97E-10 | 0.000120554 | 0.000249603 | 0.630001 |
| rs2071647 | A | T | 0.2755 | 0.1526 | 0.0258 | 3.30E-09 | -0.000308185 | 0.000252225 | 0.22 |
| rs2144013 | G | A | 0.2093 | 0.2234 | 0.0317 | 1.76E-12 | -0.00034273 | 0.000277634 | 0.22 |
| rs2269247 | T | C | 0.1804 | 0.1709 | 0.0295 | 7.28E-09 | -1.41E-05 | 0.000290554 | 0.96 |
| rs231971 | G | A | 0.1026 | 0.2411 | 0.0399 | 1.55E-09 | -0.000413997 | 0.000412524 | 0.32 |
| rs4566101 | C | T | 0.2697 | 0.1755 | 0.0255 | 6.23E-12 | -0.000168154 | 0.000253203 | 0.51 |
| rs506770 | C | G | 0.768 | 1.0048 | 0.0426 | 3.33E-123 | -0.000291463 | 0.000261814 | 0.27 |
| rs55996894 | C | G | 0.204 | -0.1785 | 0.0323 | 3.13E-08 | 0.000280107 | 0.000294291 | 0.34 |
| rs62410259 | A | G | 0.077 | -0.3796 | 0.0533 | 1.02E-12 | -6.40E-05 | 0.000427407 | 0.88 |
| rs689 | T | A | 0.7109 | 0.7004 | 0.0354 | 2.30E-87 | 0.000101864 | 0.000248823 | 0.68 |
| rs6909461 | C | A | 0.2529 | -0.314 | 0.0332 | 3.06E-21 | 0.000395811 | 0.000266845 | 0.14 |
| rs741172 | T | C | 0.3205 | -0.2034 | 0.0258 | 3.11E-15 | -0.00021081 | 0.000240287 | 0.38 |
| rs79075295 | A | G | 0.0921 | -0.4192 | 0.0621 | 1.46E-11 | 0.000249796 | 0.000643013 | 0.7 |
| rs8056814 | A | G | 0.0793 | 0.2641 | 0.0415 | 1.99E-10 | -0.000363193 | 0.000405176 | 0.37 |
| rs9468618 | T | C | 0.0975 | -0.3009 | 0.0489 | 7.53E-10 | -0.00031478 | 0.0003921 | 0.42 |

SNP, single nucleotide polymorphism; se, standard error; pval, p-value; UTIs, urinary tract infections.

**Table S9** The checklist based on the work of Woolf et al.

| Question | Criteria | | Available to all users (Y), partial coverage (P), not provided by MR-Base (N), item not analysis related (NA) |
| --- | --- | --- | --- |
| Clear articulation of the research question |  | |  |
| 1) Clearly define exposure(s) | State clearly what the exposure is | | Y |
| 2) Clearly define outcome(s) | Clearly state what the outcome is | | Y |
| 3) State how many exposure/outcome relationships were tested in the main analysis | State how many exposure/outcome relationships were tested | | Y |
| 4) Clearly state the hypothesis under investigation | State the hypothesis under investigation | | Y |
| Data sources |  | |  |
| 5) Provide an evidence trail to a description of the source GWAS^a^ | e.g., a citation or link to relevant study or methods papers. | | Y |
| 6) Describe the methods used to recruit participants into GWAS^a^ | As in question | | P |
| 7) State the number of participants included in GWAS^a^ | As in question | | Y |
| 8) Describe how exposure and outcome were measured^a^ | As in question | | N |
| 9) State the units that the exposure and outcome were measured in and whether the data for these measures were transformed (or if the study is a case/control)^a^ | State the units of the GWAS or that it was a case/control | | Y |
| 10) List covariates included in GWAS | As in question | | P |
| 11) Describe quality control procedures adopted in GWAS or explicitly provide a reference to it^a^ | Provide some information on the GWAS QC. | | P |
| 12) If applicable, comment on any attempts made to address inadequate quality control or GWAS design in MR analysis^a^ | If they describe the QC, do they describe any attempts to improve it | | N |
| 13) For MR studies examining where exposure and/or outcome is a disease/binary trait, describe if cases were incident or prevalent or both | As in question | | N |
| 2SMR specific assumptions |  | |  |
| 14) Provide adequate descriptive information on GWAS samples to assess whether they represent the same underlying population | e.g., at least two of age, sex, and ancestry being similar, or dissimilarity on one of the domains | | Y |
| 15) Provide information on sample overlap |  | | Y |
| Data harmonization |  | |  |
| 16) Explain how reference alleles were harmonized across exposure and outcome datasets |  | | Y |
| 17) Describe how palindromic SNPs were addressed | |  | Y |
| Instrument construction |  | |  |
| 18) Provide clear criteria or a clear description for the inclusion of genetic variants as instruments | e.g., a biological justification or GWAS P-value threshold | | Y |
| 19) Describe whether genetic variant-exposure and genetic variant-outcome association estimates were obtained from a discovery GWAS sample, replication GWAS sample, pooled discovery-replication GWAS samples or a meta-analysis of two or more samples, or another source |  | | Y |
| 20) Describe whether the instrument was restricted to independent variants or whether the instrument consisted of correlated variants |  | | Y |
| 21) If the instrument consisted of independent variants, how was independence defined/what were the clumping parameters used? | e.g. the LD r2, base distance | | Y |
| 22) If the instrument consisted of correlated variants, explain how this was accounted for in the model employed |  | | Y |
| 23) State the number of primary instruments constructed | e.g. the number of PGRs or distinct SNP aggregation. | | Y |
| 24) State the number of included genetic variants. | e.g. the number of SNPs | | Y |
| Instrumental variable (IV) assumptions and considerations |  | |  |
| 25) Describe how IV assumption 1 (relevance) was assessed | i.e., provide an r2 or F statistic for each SNP or the PGRS | | Y |
| 26) Describe how IV assumption 2 (independence) was assessed | i.e., do they describe methods for ensuring that instruments are not associated with confounders, like using BOLT LMM, adjusting for ancestry/PCs, within family GWASs, etc | | Y |
| 27) Describe how IV assumption 3 (exclusion restriction) was assessed | e.g. stating the use of a falsification test | | Y |
| 28) Describe how homogeneity/monotonicity/constant effect (IV assumption 4) was assessed |  | | P |
| 29) If applicable, acknowledge all major assumptions introduced by falsification tests | e.g. the INSIDE assumption for MR-Egger | | Y |
| 30) Explain why the used sensitivity analyses were used | e.g. because they allow the relaxation of an assumption | | Y |
| Analytic methods |  | |  |
| 31) List the primary model(s) employed to examine the exposure-outcome association | e.g. inverse variance weighted model or a Wald ratio | | Y |
| 32) Describe whether any proxy genetic variants were used in the analysis, and if so which reference panels and LD threshold criteria were used for selecting these proxy genetic variants, and how these were derived (e.g. SNP SNAP, LD Link, MR-Base, manual look up, etc) | NB: if no description was provided, then papers were marked as providing insufficient information. This is because the default in MR-Base web platform is to try to use proxies, but not state if they were used. Without any information provided it is impossible for a reader to know if the paper used the default settings or chose not to use proxies | | Y |
| 33) Describe whether a power calculation has been performed. |  | | N |
| 34) If there was >1 exposure/outcome relationships, either state whether this was corrected for multiple testing or provide justification for/against correcting for multiple testing (or discuss in discussion) |  | | Y |
| 35) Describe any attempts to examine directionality of genetic variant-exposure and genetic variant-outcome associations | e.g. the use of a bidirectional design or Steiger filtering. | | N |
| 36) If analyses were performed using a single genetic variant as an instrumental variable, was co-localisation performed |  | | NA |
| 37) If the exposure GWAS used a binary variable, is the causal effect described in terms of liability (or susceptibility) to the exposure in the discussion |  | | Y |
| 38) Describe the causal effect in terms of the units of measurement, or relate it to a clinically understandable scale of the exposure | NB: for measures like psychometric instruments which do not have ‘units’, a description of the effect in terms of a standard deviation is sufficient. Other standardized measures require the standard deviation to be defined in terms of units | | Y |
| 39) Describe whether any plots are presented to visualize results | NB: only plots used to visualize MR results are eligible | | Y |
| 40) Describe whether other forms of MR specific bias could be present in analyses | e.g. dynastic effects, assortative mating, canalization. NB:. if pleiotropy or another bias is covered elsewhere in the text, only residual pleiotropy/bias would be eligible | | Y |
| Reproducibility and open science |  | |  |
| 41) Present all data used to perform all analyses or describe where data can be accessed | e.g. if using data from another study, provide citations; if using primary data provide link to data access | | Y |
| 42) Provide R code for performing all analyses |  | | N |

MR, Mendelian randomization; LD, linkage disequilibrium; GWAS, genome-wide association study; QC, quality control; PGRs, polygenic risk score; SNP, single nucleotide polymorphism; PCs, principal components (of the genetic relationship matrix); IV, instrumental variable.

^a^Give information separately for exposure and outcome GWAS.
